# Escherichia coli K-12 Lacks a High-Affinity Assimilatory Cysteine Importer

**DOI:** 10.1128/mBio.01073-20

**Published:** 2020-06-09

**Authors:** Yidan Zhou, James A. Imlay

**Affiliations:** a Department of Microbiology, University of Illinois, Urbana, Illinois, USA; New York University School of Medicine

**Keywords:** LIV system, YhaO, amino acid uptake

## Abstract

The most direct route by which microbes might assimilate sulfur would be by importing cysteine. However, alone among the amino acids, cysteine does not have well-characterized importers. We determined that Escherichia coli can rapidly import cysteine, but in our experiments, it did so primarily through the LIV ATP-driven system that is dedicated to branched-chain amino acids. The affinity of this system for cysteine is far lower than for Leu, Ile, and Val, and so in their presence, cysteine is excluded. Thus, this transport is unlikely to be relevant in natural environments. Growth studies, transcriptomics, and transport assays failed to detect any high-affinity importer that is dedicated to cysteine assimilation. Enteric bacteria do not contain the putative cysteine importer that was identified in Campylobacter jejuni. This situation is surprising, because E. coli deploys ion- and/or ATP-driven transporters that import cystine, the oxidized form of cysteine, with high affinity and specificity. We conjecture that in oxic environments, molecular oxygen oxidizes environmental cysteine to cystine, which E. coli imports. In anoxic environments where cysteine is stable, the cell chooses to assimilate hydrogen sulfide instead. Calculations suggest that this alternative is almost as economical, and it avoids the toxic effects that can result when excess cysteine enters the cell.

## Abstract (precis)

This investigation discovered that Escherichia coli lacks a transporter dedicated to the assimilation of cysteine, an outcome that is in striking contrast to the many transporters devoted to the other 19 amino acids. We ascribe the lack of a high-affinity cysteine importer to two considerations. First, the chemical reactivity of this amino acid is unique, and its poorly controlled import can have adverse consequences for the cell. Second, our analysis suggests that the economics of biosynthesis depend sharply upon whether the cell is respiring or fermenting. In the anoxic habitats in which cysteine might be found, the value of import versus biosynthesis is strongly reduced compared to that in oxic habitats. These studies may explain why bacteria choose to synthesize rather than to import other useful biomolecules as well.

## INTRODUCTION

Sulfur owes its place in biochemistry to several singular features: its ability to access cationic, neutral radical, and anionic redox states at physiological potentials; its nucleophilicity, which leverages a pK_a_ near neutrality; and its tendency to avidly bind transition metals (1). These properties are exploited in structural and catalytic disulfide bonds, radical reaction mechanisms, the formation of energetic thioester species, and the coordination of active-site transition metals. Sulfur atoms participate in these activities not only in the guise of the cysteine and methionine residues of proteins but also via a substantial number of specialized cofactors: iron-sulfur clusters, molybdopterin, pantothenate, lipoate, thiamine, biotin, and glutathione. Escherichia coli contains 130 mM sulfur atoms (2), and so growing cells must find ways to continuously import sulfur at a high rate.

Primordial cells evolved in a reducing environment (3), and it is likely that they directly incorporated ambient hydrogen sulfide into various sulfur-containing biomolecules. This strategy persists in a minority of archaea that live in anoxic sulfidic habitats (4–7). However, most contemporary organisms feature a two-stage sulfur-trafficking scheme with free cysteine at its hub. They can import a wide variety of sulfur compounds and convert them to cysteine; then, a cohort of enzymes transfer sulfur atoms from this free cysteine pool to the various biomolecules that require them. Allosteric and transcriptional regulatory devices collaborate to ensure that the cysteine pool remains adequate. For example, if a slowdown in sulfur acquisition causes the cysteine pool in Escherichia coli to shrink, feedback control is loosened upon the activity of *O*-acetylserine synthase, the first committed step in cysteine synthesis (8). The deficit is also indirectly relayed to the transcription factor CysB. Activated CysB then induces the transporters that import various sulfur species and the enzymes that convert them to cysteine (9).

Molecular oxygen chemically oxidizes hydrogen sulfide, and so in oxic environments, bacteria encounter inorganic sulfur in the form of sulfate instead. This species must first be reduced back to hydrogen sulfide before it can be incorporated into cysteine. Eight electrons are required, and the net energy cost is large. However, E. coli is a heterotroph that also encounters amino acids from degraded biomass. The released cysteine spontaneously reacts with oxygen to form cystine (10, 11). Thus, an economical alternative to sulfate assimilation is for the bacterium to import cystine. In fact, most bacteria have high-affinity cystine importers, and the CysB system of E. coli prioritizes cystine as its cellular sulfur source (2). This strategy does have a drawback: when environmental concentrations of cystine fluctuate, its overimport can temporarily drive intracellular cysteine pools to levels that disrupt metabolism (11, 12).

In anoxic environments, extracellular cysteine remains in its reduced form, and we anticipated that anaerobes and facultative bacteria, including E. coli, would similarly favor it as a sulfur source. However, the actual situation is murky. Only a single bacterial cysteine importer, YhaO, has been functionally studied, e.g., in the fish pathogen Yersinia ruckeri and in the mouse pathogen Salmonella enterica serovar Typhimurium (12, 13). (YhaO has also been denoted CyuP.) YhaO was shown to be an importer that is driven by the proton motive force (14). However, *yhaO* lies in an operon with a second gene, *yhaM*, that encodes a cysteine desulfidase that degrades cysteine to pyruvate, ammonium, and hydrogen sulfide (13, 15, 16). The sulfide passively diffuses out of the cell (17). The arrangement suggests that its physiological purpose is to degrade cysteine as a carbon or nitrogen source rather than to assimilate it. The same *yhaOM* operon is found in E. coli.

One other potential cysteine importer has been identified among the bacteria. Independent groups isolated a periplasmic binding protein from Campylobacter jejuni and Neisseria gonorrhoeae that copurified with cysteine and that selectively bound it with nanomolar avidity (18, 19). The gene encoding the binding protein is adjacent to those for recognizable members of an ABC-type import system, and so it seems likely, albeit unproven, that these proteins comprise an authentic high-affinity cysteine import system. Yet, these genes are not abundant in the bacterial kingdom. Homologs are not evident either in E. coli or in other enterics.

Our ignorance about cysteine transport stands in contrast to our knowledge about the other nineteen amino acids, for which importers are well characterized (Table 1; see also Table S1 and Fig. S1 in the supplemental material). One problem may be technical: in standard oxic lab media, cysteine readily oxidizes to cystine, and consequent cystine import can interfere with screens for cysteine uptake. Cystine may also contaminate cysteine that is purchased from chemical suppliers. To avoid these problems, in this study, we probed cysteine import into E. coli under anoxic conditions using mutants that lack its cystine importers.

**TABLE 1:** High-affinity l-amino acid importers of E. coli K-12^*a*^

Amino acid	Importer category	
	ABC-type	Ion-driven
Alanine		✓
Arginine	✓	✓
Asparagine		✓
Aspartic acid	✓	✓
Cystine	✓	✓
Glycine		✓
Glutamine	✓	
Glutamic acid	✓	✓
Histidine	✓	
Isoleucine	✓	✓
Leucine	✓	✓
Lysine	✓	✓
Methionine	✓	
Phenylalanine	✓	✓
Proline		✓
Serine		✓
Threonine		✓
Tryptophan		✓
Tyrosine		✓
Valine	✓	✓
Cysteine^*b*^		

a Importers are categorized as ATP-binding cassette (ABC) transporters and ion-driven transporters. Data are collected from EcoCyc. The importers are identified by name in Table S1 in the supplemental material.

b Uniquely, cysteine is not known to have a dedicated high-affinity importer.

10.1128/mBio.01073-20.1 FIG S1 Affinities of E. coli l-amino acid importers for their cognate substrates. Note the scale change between panels A and B. Data are drawn from EcoCyc (https://ecocyc.org/) and references therein. ABC-type transporters shown here are ArgT-HisQPM (Arg), ArtJIMQP (Arg), GltIKJL (Asp), TcyJLN (cystine), GlnQPH (Gln), GltIKJL (Glu), HisJQPM (His), LivFGHMK/J (Ile, Leu, and Val), MetINQ (Met), and LivFGHMK (Phe). Secondary (ion-driven) transporters include CycA (Ala and Gly), TcyP (cystine), GlpP (Glu and Asp), GltS (Glu), LysP (Lys), PheP (Phe), BrnQ (Ile), AroP (Phe, Trp, and Tyr), PutP (Pro), TdcC (Thr), SdaC (Ser), SstT (Ser), and Mtr (Trp). DcuA (*K_m_* = 43 μM) and DctA (*K_m_* = 148 μM) were for use of Asp as a nitrogen and carbon source, respectively, and TnaB (*K_m_* = 70 μM) was for use of Trp as carbon source (not shown). Binding constants have not yet been reported for other transporters. *K_D_* and *K_m_* are not necessarily equivalent measures of substrate binding. Download FIG S1, PDF file, 0.2 MB. Copyright © 2020 Zhou and Imlay. 2020 Zhou and Imlay This content is distributed under the terms of the Creative Commons Attribution 4.0 International license.

10.1128/mBio.01073-20.8 TABLE S1 Known high-affinity importers of l-amino acids in E. coli. Download Table S1, DOCX file, 0.1 MB. Copyright © 2020 Zhou and Imlay. 2020 Zhou and Imlay This content is distributed under the terms of the Creative Commons Attribution 4.0 International license.

Surprisingly, we found that cysteine primarily enters E. coli through transporters that are dedicated to other amino acids. Their affinity for cysteine is relatively low, and the other amino acids easily blocked cysteine uptake. Since E. coli seems likely to encounter cysteine only when other amino acids are present, we conclude that E. coli lacks an effective system for assimilatory cysteine import. We speculate that in anoxic environments, E. coli uses hydrogen sulfide as its primary sulfur source. If so, the modest energetic savings that might be recouped from using cysteine rather than sulfide may be outweighed by the risks associated with cysteine overimport.

## RESULTS

### The import of cysteine into E. coli is inefficient if other amino acids are present.

**FIG 1:**
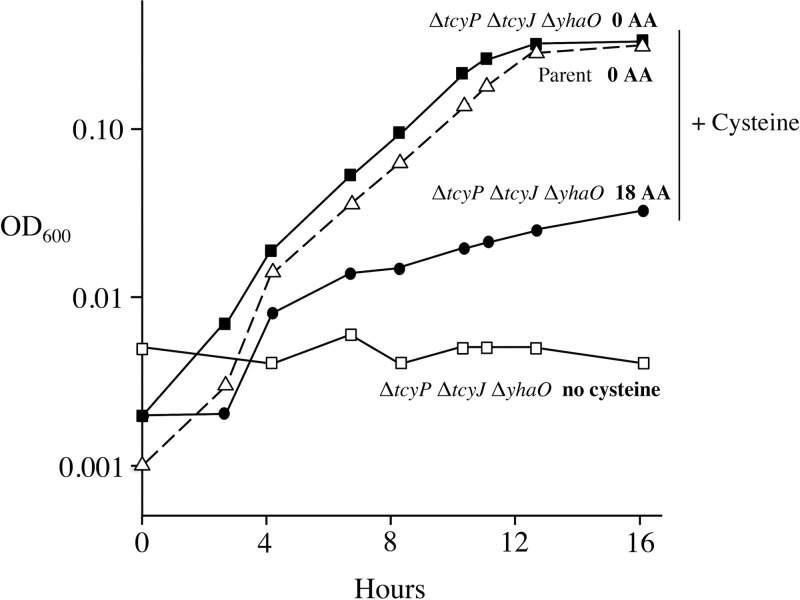
E. coli can use cysteine as its sole sulfur source. Strains lacking the capacity to assimilate sulfate (Δ*cysA* Δ*cysJIH*) were cultured under anoxic conditions with 40 μM cysteine. Strains that additionally lacked the importer associated with cysteine degradation (Δ*yhaO*) and importers of cystine (Δ*tcyP* Δ*tcyJ*) grew similarly to the parent strain (dashed line). Where indicated, 100 μM of the 18 nonsulfurous amino acids was provided. The data are representative of three independent experiments. Strain, ZYD114 (Δ*tcyP* Δ*tcyJ* Δ*yhaO* Δ*cysA* Δ*cysJIH*); parent strain, KCI826 (Δ*cysJIH* Δ*cysA751*).

To test whether cells can utilize cysteine as a sulfur source, we prepared a medium that was depleted of adventitious sulfate (see Materials and Methods), and we employed a strain that lacks sulfate assimilatory enzymes (Δ*cysA* Δ*cysJIH*). These mutations avoid problems that might arise if sulfate contaminates supplements. Such a strain did not grow at all in our medium without the deliberate addition of an additional sulfur source. Cysteine is easily oxidized by molecular oxygen into cystine, which creates a problem for studying cysteine import in oxic media. Therefore, growth experiments were performed in an anaerobic chamber. Using these precautions, we observed that E. coli indeed was able to use moderate concentrations of cysteine as its sole sulfur source (Fig. 1). Thus, the bacterium possesses at least one cysteine importer.

We employed RNA sequencing in order to identify a dedicated cysteine importer. The known sulfur assimilatory genes are strongly induced when E. coli grows on sulfate, which is a relatively poor and uneconomical sulfur source, and they are sharply repressed when cystine is provided (8, 20). Table 2 confirmed that pattern. However, these data did not reveal any novel transport proteins that might be good candidates to be dedicated cysteine importers. YhaO has been proposed to be a cysteine importer (12). However, several of its features do not follow the pattern of sulfur-assimilatory proteins. As mentioned, *yhaO* shares an operon with *yhaM*, which encodes cysteine-degrading enzyme that is more likely to reflect a catabolic purpose. Second, consistent with its induction by high levels of exogenous cysteine (13, 21), the transcriptomics data revealed that YhaO was poorly expressed in sulfate medium, and it was induced when cystine was provided (Table 2). Cystine import causes the intracellular accumulation of excess cysteine (11). Such a regulatory pattern would fit a catabolic function, but it contrasts with most sulfur import proteins, which are highly expressed in sulfate medium, in order to scavenge better sulfur sources. Finally, sulfur importers have notably few cysteine residues, presumably so they can easily be translated when intracellular cysteine pools are low (2). In contrast, E. coli YhaO and YhaM are relatively cysteine rich (Table 2).

**TABLE 2:** Responses of selected transcripts to the import of cystine^*a*^

Name	Fold change	Function	Cysteine content (%)
*yhaO*	130	Putative cysteine importer	1.1
*cysP*	−94	Thiosulfate binding protein	0
*cysU*	−62	Sulfate/thiosulfate importer	0.7
*yhaM*	56	Putative cysteine desulfidase	1.7
*tcyP*	−37	Cystine importer	0.2
*livJ*	−7	BCAA transporter subunit	0.6
*livK*	−3	Leucine transporter subunit	
*livH*	−4	BCAA transporter subunit	
*livM*	−4	BCAA transporter subunit	
*livG*	−5	BCAA transporter subunit	
*livF*	−5	BCAA transporter subunit	
*brnQ*	−1	BCAA transporter	1.1
*tcyJ*	−2	Cystine importer binding protein	0
*tcyL*	−1	Cystine importer subunit	
*tcyN*	−1	Cystine importer subunit	
*cycA*	2	Alanine/serine/glycine/cycloserine transporter	1.3
*yaaJ*	2	Putative alanine/glycine transporter	1.7

a Sulfate-grown cells were exposed to 50 μM cystine for 10 min, and RNA sequence analysis was performed. The full data set can be found in reference 11. Gray shading indicates genes known to be regulated by the transcriptional activator CysB. The cysteine content of CysB-regulated proteins averages 0.2% compared to 1.7% for the entire genome and 0.8% for amino acid importers.

In fact, when these genes were knocked out, E. coli remained capable of good growth with cysteine. To ensure that the experiment was not marred by cystine contaminating the cysteine, the experiment was reproduced in a strain that additionally lacked the two cystine importers (TcyP and TcyJLN). The Δ*yhaO* Δ*tcyP* Δ*tcyJLN* strain grew as well as its YhaO^+^ Tcy^+^ parent when provided cysteine as the sole sulfur source (Fig. 1). This triple mutant was used as the parental strain in subsequent experiments unless otherwise noted.

The natural circumstance in which E. coli will encounter cysteine is presumably when nearby organisms have died and their proteins have degraded. In this situation, E. coli will also encounter other amino acids. Strikingly, when the standard 18 l-amino acids (aa) (excluding cysteine and methionine) were included in the growth experiment, the Δ*yhaO* Δ*tcyP* Δ*tcyJIN* strain substantially lost the ability to use cysteine (Fig. 1). This behavior does not match that of the other amino acids: in separate tests, we confirmed that the other amino acid auxotrophs that were readily available in the lab were able to be effectively supplemented with the necessary amino acid even when the other amino acids were present in a similar 2.5-fold excess (Pro, His, Thr, Leu, Arg, Phe, Trp, Tyr, Met, Asp, Glu, Gln, Pro, Ile, and Lys) (data not shown). This result suggested that E. coli might lack a dedicated cysteine importer and that cysteine might be imported artifactually through a transporter with a much higher affinity for a different amino acid.

### Cysteine treatment sensitizes cells to H_2_O_2_.

Our lab identified the high-affinity cystine importer TcyP in E. coli by exploiting the ability of intracellular cysteine to sensitize cells to H_2_O_2_ (2). When cystine enters the cell, it is reduced to cysteine. High levels of intracellular cysteine potentiate oxidative DNA damage by driving the Fenton reaction, by recycling Fe^3+^ to Fe^2+^ (22–25):

Fe2++H2O2→Fe3++OH−+HO·

RSH+Fe3+→RS·+Fe2+

**FIG 2:**
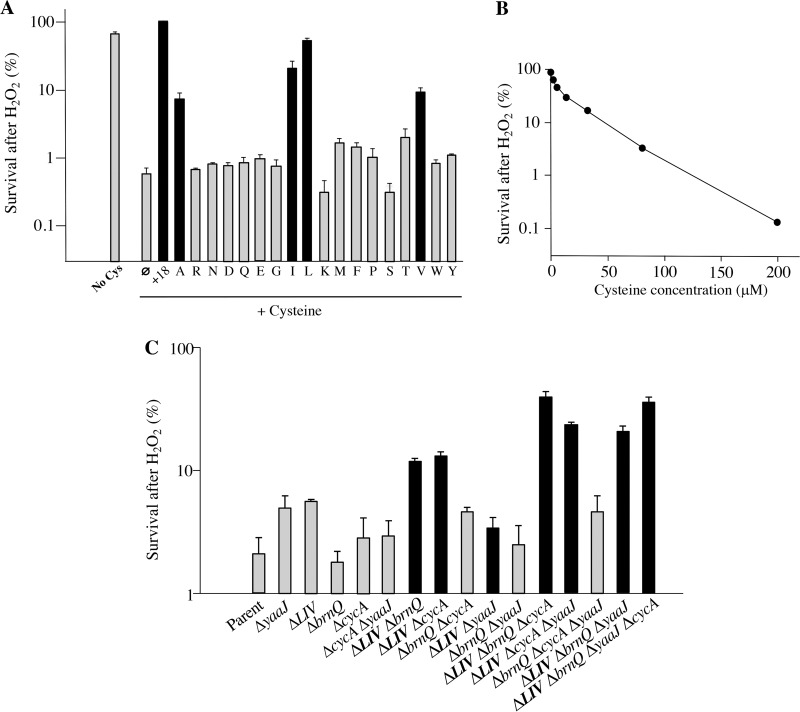
Branched-chain amino acids prevent cysteine from augmenting H_2_O_2_ toxicity. (A) Cells growing in oxic sulfate medium were briefly exposed to 3 min of 200 μM cysteine and 2.5 mM H_2_O_2_ for 4 min, as described in Materials and Methods. DTPA was added to suppress cysteine oxidation. Cysteine greatly stimulated cell killing (first and second bars). Where indicated, 500 μM other amino acids was added along with cysteine. Leucine, isoleucine, valine, and alanine had strong protective effects, as highlighted by black bars. Strain, ZYD15 (Δ*tcyP* Δ*tcyJ* Δ*yhaO*). (B) High cysteine concentrations maximize cell killing by H_2_O_2_. (C) The branched-chain and alanine importers were deleted individually or in concert from ZYD15, and these mutants were exposed to 200 μM cysteine plus H_2_O_2_. The LIV system and BrnQ are ABC-type and ion-driven branched-chain importers, respectively, while CycA and YaaJ are proposed alanine importers. Mutants lacking the LIV system exhibited greater resistance. Error bars represent standard errors of the means (SEMs) from three independent experiments.

The hydroxyl radical (HO·) is a powerful oxidant that oxidizes most biomolecules upon contact. If HO· is generated in the vicinity of DNA, it can create DNA lesions and lead to cell death. In the previous work, our selection for mutants resistant to the combination of cystine and H_2_O_2_ yielded *tcyP* mutants that do not import cystine (2). Here, a similar approach was taken to isolate mutants lacking a cysteine importer. When the parent strain (Δ*tcyP* Δ*tcyJIN* Δ*yhaO*) was grown in minimal medium lacking amino acids and then exposed to cysteine and H_2_O_2_, >99% of cells were killed (Fig. 2A, first two bars). This result was comparable to that seen with cystine. However, whereas only a few micromolar cystine is necessary to cause sensitivity, in accord with the 2 μM *K_m_* value of the TcyP cystine importer (2), relatively high cysteine concentrations were necessary for extensive cell death (Fig. 2B). This observation suggested that cysteine entered the cell through a transporter with a relatively low affinity for it. Killing did not occur if 18 aa were added immediately before the cysteine exposure (Fig. 2A, third bar). We speculated that the resistance caused by 18 aa was due to amino acid transport blockage and that the cysteine enters the cell through an importer of other amino acids. This idea would be consistent with the growth data shown in Fig. 1. Any downregulation of the transporters was not relevant, because the 18 aa were added immediately before the cysteine. Therefore, their impact had to occur via inhibition of the extant transporters.

We generated ∼10,000 transposon mutants and selected for isolates that could survive repeated rounds of cysteine/H_2_O_2_ exposure (see Materials and Methods). Two mutations were isolated that substantially diminished rates of killing; they both lay in the *clpPX* operon and did not involve inner membrane proteins that could conceivably transport proteins. (The basis of this *clpPX* phenotype is explored in another study.) Our failure to recover mutations in any plausible transporter may indicate that multiple transporters can contribute to cysteine import.

As an alternative approach, we supplemented the medium with 18 amino acids individually in order to identify those that could block cysteine/H_2_O_2_ killing (Fig. 2A). Leucine, isoleucine, valine, and alanine provided the greatest protection. E. coli possesses two dedicated alanine importers, YaaJ and CycA (26, 27), and two branched-chain amino acid (BCAA) importers, LivJ/LivKHMGF (denoted here as the LIV system) and BrnQ (28, 29). These four transporters were knocked out individually and in groups (Fig. 2C). A mutant lacking all four systems was strongly resistant, but this degree of protection was not recapitulated by any one single mutant. The data from the mutant combinations suggest that the LIV system had the largest impact upon cysteine/H_2_O_2_ sensitivity and thus putatively upon cysteine import. The BrnQ, CycA, and YaaJ transporters each had relatively minor, albeit nonzero, contributions. We infer that each of these importers has some ability to import cysteine when it is present at high concentrations and competing transporter substrates are absent. The side chain of cysteine is small and is regarded as minimally polar (30, 31), and so its ability to serve as a substrate for branched-chain transporters is not wholly surprising.

### Cysteine is a poor substrate for the LIV system.

**FIG 3:**
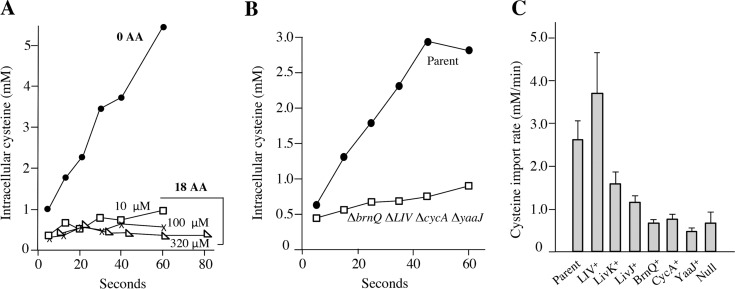
Cysteine is imported primarily through the LIV system. (A) The import of 20 μM [^14^C]cysteine was monitored upon its addition to sulfate-grown cells. Where indicated, the 18 nonsulfurous aa were included at the specified concentrations. Chloramphenicol was included to block protein synthesis. The data are representative of three independent experiments. Strain, ZYD15 (Δ*tcyP* Δ*tcyJ* Δ*yhaO)*. (B) Transport measurements were conducted with 20 μM [^14^C]cysteine in medium lacking other amino acids. The data are representative of three independent experiments. Strains, ZYD15 (Δ*tcyP* Δ*tcyJ* Δ*yhaO*) and ZYD138 [Δ*tcyP* Δ*tcyJ* Δ*yhaO* Δ*brnQ* Δ*liv*(*KMGHF*) Δ*yaaJ* Δ*cycA*]. (C) Transport measurements with 20 μM cysteine were conducted using mutants that each express only a single candidate importer. In accord with the H_2_O_2_ experiments, only the LIV-expressing strain imported cysteine at a rate comparable to that of the parent strain. LivK and LivJ are the two alternative periplasmic binding proteins that LIV employs, and their contributions were measured in strains expressing the membrane-bound components of the LIV system. Error bars represent SEMs from three independent experiments. Strains, ZYD15, ZYD214, ZYD180, ZYD134, ZYD119, and ZYD138.

Most bacterial amino acid transporters exhibit *K_m_* values for their substrates in the range of 0.1 to 5 μM (see Fig. S1 in the supplemental material). It is likely that this range represents the concentrations of amino acids that cells encounter in nature. Transport studies were performed at cysteine concentrations that approached these values. In the absence of other amino acids, cells were able to import 20 μM cysteine at a rate (4.2 mM/min) that is sufficient to satisfy the maximum sulfur requirement (2.2 mM/min [2]) (Fig. 3A). This result explains why cells grew well as shown in Fig. 1. However, the addition of 18 aa blocked cysteine import completely. This result paralleled the results observed in the H_2_O_2_ experiments. Indeed, the Δ*LIV* Δ*brnQ* Δ*cycA* Δ*yaaJ* mutations collectively suppressed import (Fig. 3B). Accordingly, this null mutant grew slowly when only cysteine was provided as the sole sulfur source (see Fig. S2); the residual growth was inhibited by other amino acids, suggesting that it depended upon persistent cysteine leakage into the cell through other unidentified amino acid transporters. Cysteine transport was then measured in strains that expressed only CycA, LIV, YaaJ, or BrnQ in the Δ*tcyP* Δ*tcyJIN* Δ*yhaO* background. The LIV system was the primary cysteine transporter, and its cysteine transport rate was comparable to that of the parent strain (Fig. 3C).

10.1128/mBio.01073-20.2 FIG S2 Cysteine supports only slow growth when branched-chain and alanine importers are absent. Cells were grown in anoxic medium. Where indicated, medium contained 40 μM cysteine and 100 μM of the 18 nonsulfurous amino acids. The residual cysteine import is impaired by competing amino acids, suggesting that the slow persistent cysteine import may occur through still other transporters dedicated to other amino acids. Strains: ZYD114 and ZYD210. Δ*null* represents a strain lacking the branched-chain and alanine importers [Δ*brnQ* Δ*liv*(*KMGHF*) Δ*yaaJ* Δ*cycA*]. Download FIG S2, PDF file, 0.2 MB. Copyright © 2020 Zhou and Imlay. 2020 Zhou and Imlay This content is distributed under the terms of the Creative Commons Attribution 4.0 International license.

Is the LIV system fully competent as a cysteine transporter? The pattern of regulation suggests that it has not evolved to play that role. Sulfur import proteins are invariably activated by the CysB transcription factor, whereas branched-chain metabolism—including the *livJKHMGF* genes—is controlled by Lrp (32, 33). Cystine addition has a potent effect on the CysB regulon, as the consequent accumulation of intracellular cysteine turned down the expression of sulfur acquisition genes by more than 30-fold (Table 2). In contrast, the *livJKHMGF* genes were repressed only slightly, 2.9- to 6.6-fold, which accords with the moderate effect that intracellular cysteine exerts on the Lrp system (11). Conversely, transcriptional fusions showed that the *livJ* and *livK* promoters were induced 22-fold and 12-fold, respectively, by the absence of leucine (32). Therefore, the regulatory data indicate that the LIV system is relatively poorly responsive to the sulfur needs of the cell. We also noted that the cysteine content (Table 2) of the LIV proteins is higher than that of most dedicated sulfur acquisition proteins.

**FIG 4:**
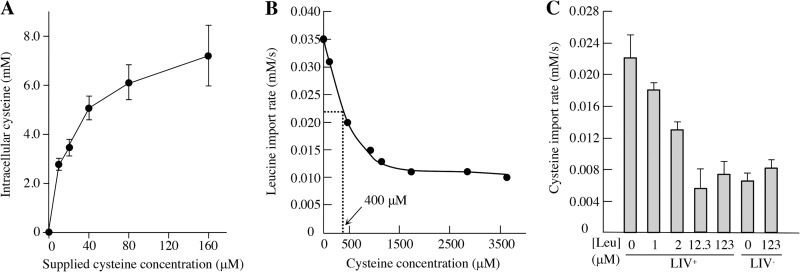
The LIV system binds cysteine with low affinity. (A) Cysteine transport measurements were determined for a strain expressing the LIV system. Data represent the 93-s time point and indicate an apparent *K_m_* for cysteine of approximately 20 μM. For comparison, the apparent *K_m_* for leucine binding by LivJ and by LivK is 0.4 μM (34). Error bars show SEMs from three technical replicates. Strain, ZYD214 (Δ*tcyP* Δ*tcyJ* Δ*yhaO* Δ*cycA* Δ*brnQ* Δ*yaaJ*) (B) The import of 20 μM radiolabeled leucine was determined in the presence of various concentrations of cold cysteine in a LIV-proficient strain. A 20-fold higher concentration of cysteine (400 μM) was needed to slow leucine import by one-half. The minor leucine transport that persists at high cysteine concentrations is likely performed by an unknown transporter (see Results). Strain, ZYD214. (C). The import of 20 μM [^14^C]cysteine was measured in LIV-proficient and LIV-deficient strains in the presence of increasing concentrations of leucine. The LIV-deficient strain indicated slight residual cysteine import by an unknown Leu-insensitive route. Strains, ZYD214 and ZYD138.

Finally, we examined the kinetics of cysteine import (Fig. 4A). In the absence of amino acids, the half-maximum rate of cysteine transport was observed at approximately 20 μM cysteine, whereas the measured equilibrium dissociation constant (*K_D_*) values for leucine have been measured to be 0.4 μM (34). This large difference was confirmed in competitive transport assays. Approximately 400 μM cysteine was needed to reduce by 50% the import of 20 μM leucine through the branched-chain transporters (Fig. 4B). Conversely, 2 μM leucine sufficed to similarly inhibit the import of 20 μM cysteine, and all of the Leu-sensitive import was blocked by 12 μM (Fig. 4C). In sum, these data show that the LIV system has too low an avidity for cysteine to serve as a useful import system when cysteine concentrations are small or when competing amino acids are present. Both of these conditions are likely to apply to natural environments.

### TcyP and TcyJLN do not transport cysteine.

**FIG 5:**
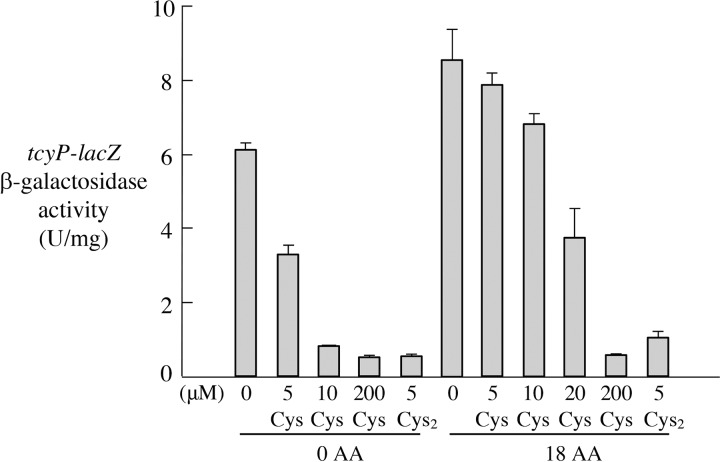
Competing amino acids prevent natural concentrations of extracellular cysteine from boosting the intracellular cysteine pools. Cells carrying a *tcyP-lacZ* were grown with various amounts of cysteine (Cys) or cystine (Cys_2_). Concentrations are noted in the figure. β-Galactosidase activity was measured. Expression of *tcyP*, encoding a cystine importer, is controlled by the CysB transcription factor and is turned off when the intracellular cysteine pool becomes sufficient. Where indicated, the 18 nonsulfurous aa were supplemented at 20 μM each. Error bars represent SEMs from three independent experiments. All transporters native to K-12 strains were operative in this strain (KCI1205).

The previous experiments were conducted in a Δ*tcyP* Δ*tcyJIN* Δ*yhaO* background to avoid the effects of any contaminating cystine in our radiolabeled cysteine and to circumvent YhaO induction in some of the growth protocols. We did examine the possibility that these proteins might contribute to cysteine import, despite the arguments that were presented at the outset. To do so, we used the CysB system as the native sensor of cysteine import. The CysB transcription factor is activated when the cellular cysteine pools are inadequate; among the genes that it induces is *tcyP*, encoding the ion-driven cystine importer (2). As shown in Fig. 5, low levels of cystine suffice to turn off *tcyP-lacZ*, both in the presence and in the absence of other amino acids. In contrast, higher levels of cysteine are necessary, and this repressive effect is lost for cysteine (but not cystine) when other amino acids are present. This result fits the previous data showing that transport occurs via the LIV system, and it also confirms that neither of the cystine importers nor YhaO enabled cysteine import.

### YhaO is not a sulfur acquisition transporter.

**FIG 6:**
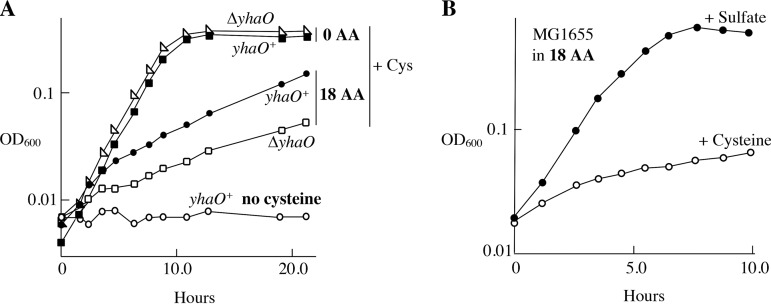
YhaO is ineffective at cysteine acquisition. (A) Growth was monitored in anoxic medium in which 40 μM cysteine was provided as the sole sulfur source. Where indicated, the medium was supplemented with 100 μM of the 18 nonsulfurous amino acids. Inhibition by competing amino acids indicates that growth depends upon cysteine entry through nonspecific transporters such as the LIV system; YhaO provides minimal assistance. The data are representative of three independent experiments. ZYD114, Δ*yhaO*; ZYD109, *yhaO^+^*. (B) Growth of the wild-type strain MG1655 was monitored in anoxic medium containing 18 aa, as in panel A. Where indicated, either sulfate or 40 μM cysteine was provided as the sole sulfur source. Similar results were obtained with another ancestral K-12 strain, W3110 (see Fig. S6 in the supplemental material).

YhaO only marginally improved cysteine utilization when cells were sulfur starved (Fig. 6A). Consequently, wild-type cells were very poor at using cysteine as a sulfur source when amino acids were present to impede cysteine import through the LIV system (Fig. 6B).

**FIG 7:**
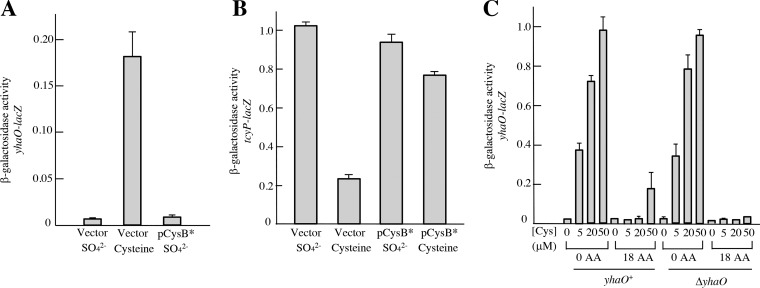
YhaO is not induced during periods of sulfur deficiency, and its induction by cysteine is dependent on nonspecific transporters. The expression of *yhaO-lacZ* and *tcyP-lacZ* transcriptional fusions were determined in sulfate media, in which cells were grown with large cysteine supplements (200 μM) for four generations. Cells were grown in anoxic medium to keep cysteine reduced. The pCysB* plasmid encodes a constitutively activated mutant form of the CysB transcription factor, which is normally activated only when cells are supplied with poor sulfur sources (e.g., sulfate). Notably, CysB activates *tcyP* expression (B) but not that of *yhaO* (A). Error bars represent the SEMs from three independent samples. Strains lacked cystine importers but retained all others. Strains, ZYD162, ZYD164, KCI1232, and KCI1234. (C) Cells were grown in anoxic sulfate medium, with or without the addition of 20 μM of the 18 nonsulfurous aa. Various concentrations of cysteine were added, and cells were cultured for one further doubling before harvesting. β-Galactosidase activity derived from a *yhaO-lacZ* fusion was measured. While cysteine import stimulated *yhaO* transcription in minimal medium, the presence of other amino acids blocked this effect, and YhaO did not facilitate its own induction. Error bars represent SEMs from three independent experiments. Strains, ZYD154, ZYD156 and SJ130.

Because YhaO is capable of cysteine import and is induced in its presence, it was puzzling that it was inadequate at sulfur scavenging. To address this point, we looked more closely at the conditions of its expression. The *yhaO-lacZ* and *tcyP-lacZ* transcriptional fusions were constructed. We observed that CysB does not induce *yhaO*, meaning that the transporter will be scant in sulfur-starved cells (Fig. 7A), while it strongly induces *tcyP* (Fig. 7B). Furthermore, YhaO was well expressed when 20 μM cysteine was provided (Fig. 7C), but this effect was completely blocked when other amino acids were present. In the latter case, not enough cysteine was imported to activate the YbaO regulator (also denoted CyuR and DecR) (13, 21, 35). These data indicate that unlike inducer-importing transporters such as LacY (36), the resting level of YhaO is not adequate to enable its own induction. Indeed, the cysteine-dependent induction of *yhaO* transcription followed the same pattern when the YhaO protein was absent. We infer that the induction of YhaO is probably triggered by the adventitious import of cysteine by the branched-chain importers, an event that is unreliable when even modest levels of branched-chain amino acids are present. This arrangement precludes the utility of YhaO as a sulfur-acquisition system, but it fits its proposed role in cysteine detoxification where a large amount of cysteine is required.

### Transporter activity upon noncognate amino acids may be common.

**FIG 8:**
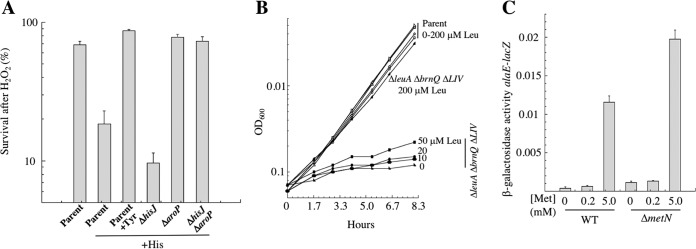
Nonspecific transporters enable entry of high concentrations of histidine, leucine, and methionine. (A) Histidine can enter the cell through AroP. Histidine amplifies sensitivity to H_2_O_2_ by mobilizing intracellular iron. Cells in minimal glucose medium were exposed to 2.5 mM H_2_O_2_ for 4 min, and survival was determined by colony formation. Where indicated, 0.5 mM histidine and/or 4 μM tyrosine was provided 3 min before H_2_O_2_ addition. Error bars represent SEMs from three independent experiments. Strains, ZYD15, ZYD30, ZYD19, and ZYD34. (B) Cells were grown in aerobic minimal medium with various amounts of leucine. Open symbols represent the parent strain, and filled symbols represent ZYD128, which lacks known leucine transporters [Δ*brnQ* Δ*liv*(*KMGHF*)] and leucine biosynthesis (Δ*leuA*). (C) Overimport of methionine activates the Lrp transcription factor, as detected by an *alaE-lacZ* translational fusion. This effect depended on high concentrations of extracellular methionine and was not diminished by elimination of the sole dedicated (MetN) methionine importer.

We initially wondered whether the ability of the LIV system to import cysteine was an unusual type of event, but in the course of this work, we encountered several other examples in which a dedicated amino acid transporter exhibited some modest activity upon noncognate substrates. First, while examining the ability of cysteine to sensitize E. coli to H_2_O_2_, we discovered that histidine did so as well (Fig. 8A). This observation turns out to have been made before (37, 38). As with cysteine, the histidine/H_2_O_2_ killing effect depends upon iron-mediated Fenton chemistry that leads to DNA damage (see Fig. S3). Because histidine is an excellent metal ligand, we suspect that excessive intracellular histidine solubilizes iron and thereby enhances its participation in Fenton chemistry. Surprisingly, the cells were not protected from histidine/H_2_O_2_ by deletion of HisJ, the periplasmic binding protein of the dedicated histidine transporter. However, tyrosine was fully protective, and we determined that histidine overimport was mediated by AroP, the high-affinity aromatic transporter (Fig. 8A). Indeed, AroP was previously reported to transport histidine with an apparent *K_m_* of 100 μM (39, 40). (Our experiments used 500 μM.) This poor affinity is likely outside the range of physiological utility, and it is in marked contrast to the submicromolar *K_m_* of AroP for its natural aromatic substrates (40).

10.1128/mBio.01073-20.3 FIG S3 Histidine-H_2_O_2_ killing is mediated by a Fenton reaction. Cells were exposed to 0.5 mM histidine for 3 min and 2.5 mM H_2_O_2_ for 4 min in the absence or presence of 1 mM dipyridyl. The stress was terminated by the addition of catalase, followed by cell dilution and plating, and surviving cells were quantified by colony formation. Dipyridyl is a cell-permeable chelator of ferrous iron that eradicates the intracellular formation of hydroxyl radicals via Fenton chemistry. Error bars represent SEMs from three independent experiments. Strain, ZYD15. Download FIG S3, PDF file, 0.1 MB. Copyright © 2020 Zhou and Imlay. 2020 Zhou and Imlay This content is distributed under the terms of the Creative Commons Attribution 4.0 International license.

Second, we constructed a Δ*leuA* Δ*brnQ* Δ*livF* strain, using LB medium that provides leucine as a component of peptides. Despite the absence of endogenous leucine synthesis and of the two leucine import systems, this strain grew well in a defined medium if >50 μM leucine was provided (Fig. 8B). The transporter was not identified, but this result is again evidence of low-affinity import through a noncognate transporter.

Finally, we observed that 5 mM methionine activated the Lrp transcription factor, but 0.2 mM methionine did not. The active dose far exceeds the *K_m_* of the dedicated methionine importer, and in fact, Lrp activation persisted in a *metN* null mutant (Fig. 8C). We infer, again, that methionine overloading likely results from its entry through a noncognate transporter.

Nonspecific, low-affinity amino acid binding by periplasmic binding proteins is common (2, 18, 41, 42). In this light, we regard the ability of LIV to import cysteine simply as another example of adventitious substrate promiscuity, and we do not believe it represents an evolved adaptation to the absence of a dedicated cysteine importer.

## DISCUSSION

Cysteine transporters have not been well characterized in bacteria. We initially suspected that this was because they had been obscured by transporters of cystine, which would be formed in aerobic culture media by the autoxidation of cysteine. We have learned that E. coli K-12 lacks a transporter dedicated to the assimilation of cysteine. Instead, low-affinity cysteine import occurs only through its accidental recognition by transporters devoted to other amino acids, and cysteine transport halts when those other amino acids are present.

E. coli propagates primarily in the colon; upon excretion, it persists for only a few days before dying or, in rare instances, being transferred to a new host (43). Free amino acids are not abundant in the colon. Virtually all dietary protein is consumed in the small intestine, and so it is the death of epithelial cells and colonic bacteria that generates much of the protein and peptides that colonic bacteria consume (44, 45). Measurements indicated that free amino acids—including cysteine—were below the 10 μM detection limit, which means their import would require high-affinity transporters (46). Notably, the few amino acids that approach millimolar levels include Leu, Val, and especially Ala, which would therefore block the low-affinity action of the E. coli LIV system upon cysteine (see Fig. S4 in the supplemental material). It follows that E. coli is unable to import cysteine in its primary habitat.

10.1128/mBio.01073-20.4 FIG S4 Alanine inhibits cysteine import through the LIV importer. LIV^+^ and LIV^−^ strains were exposed to cysteine (200 μM) in the presence or absence of alanine (500 μM) for 3 min, and then 2.5 mM H_2_O_2_ was additionally added for 4 min. The stress was terminated by the addition of catalase, followed by cell dilution and plating, and surviving cells were quantified by colony formation. Strains, ZYD214 (LIV^+^) and ZYD138 (LIV^−^). Download FIG S4, PDF file, 0.1 MB. Copyright © 2020 Zhou and Imlay. 2020 Zhou and Imlay This content is distributed under the terms of the Creative Commons Attribution 4.0 International license.

### Why does E. coli lack a cysteine importer?

The lack of high-affinity cysteine import seems odd for two reasons. First, E. coli possesses importers of all the other amino acids, which implies that cysteine is likely to be present as well. Second, the bacterium makes an impressive effort to import cystine. When intracellular cysteine dwindles, E. coli strongly induces both its TcyJLN ABC-type cystine importer and its TcyP secondary cystine importer (2). The simultaneous induction of these systems would seem to reflect the priority that E. coli places upon acquiring sulfur sources. This makes sense: the import of other amino acids may contribute to cell economy, but the elemental requirement for sulfur must be satisfied or else growth will cease.

Anoxic environments are reducing, and in the colon, cysteine seems more likely to be present than cystine. What then can one make of the absence of a cysteine importer? We favor the idea that in this habitat, E. coli assimilates sulfur from hydrogen sulfide instead. Our argument is that although in oxic environments it is far more economical to assimilate cystine than sulfate, in anoxic environments, cysteine offers little advantage over hydrogen sulfide. We calculated the ATP equivalents that are expended under oxic conditions when the cell synthesizes cysteine from sulfate versus cystine, and we performed a comparable calculation for anoxic conditions when the sulfur source is hydrogen sulfide versus cysteine. We find that under oxic conditions, the assimilation of sulfate costs the cell up to 32 ATP equivalents per cysteine formed, whereas cystine import and reduction to cysteine costs only ∼2 ATP (Table 3). The very large expense of sulfate assimilation primarily reflects the energy that would otherwise be obtained were the cell to respire the eight reducing equivalents that must be used to reduce sulfate to hydrogen sulfide. It also accounts for the ATP that would otherwise be acquired through the full oxidation of 3-phosphoglycerate, the precursor of the cysteine carbon skeleton. In contrast, only a fraction of an ATP is needed for cystine import, and only a single electron pair is required to create two cysteine moieties. In sum, the cell is strongly rewarded for employing cystine rather than sulfate as its aerobic cysteine source.

**TABLE 3:** Effective energy costs to the cell of cysteine acquisition from various sulfur sources, in oxic versus anoxic environments

Sulfur source	ATP equivalent^*a*^	
	Oxic environment	Anoxic environment
Sulfate	32	3
Cystine	2	0^*b*^
Sulfide	16^*b*^	3
Cysteine	2^*b*^	2

a Calculations are explained in Text S1A. ATP demand is rounded to the nearest whole number.

b Hydrogen sulfide and cysteine are unlikely to accumulate in fully oxic environments, due to their chemical oxidation by molecular oxygen, while cystine is likely to be reduced to cysteine by ambient sulfide in anoxic environments.

10.1128/mBio.01073-20.7 TEXT S1 (A) Calculation of the energetic cost of cysteine synthesis. (B) Calculation of the potential for the LIV system to import cysteine. (C) Calculation of the energetic cost of synthesizing an ATP-dependent cysteine importer. Download Text S1, DOCX file, 0.1 MB. Copyright © 2020 Zhou and Imlay. 2020 Zhou and Imlay This content is distributed under the terms of the Creative Commons Attribution 4.0 International license.

However, in the anoxic colon, it appears to be nearly as energy efficient for E. coli to assimilate hydrogen sulfide as to synthesize and operate a cysteine importer. Gut sulfate-reducing anaerobes drive the ambient hydrogen sulfide near the millimolar level (47–49). Hydrogen sulfide is an excellent sulfur source for E. coli; it passively diffuses across membranes and is trapped by *O*-acetylserine sulfhydrylase (CysK) to form cysteine (50, 51). Figure S5 shows that E. coli grows as quickly when using sulfide as when using cystine. Sulfide assimilation does not require the expenditure of reducing equivalents, and only a modest amount of ATP would otherwise be yielded by the fermentation of 3-phosphoglycerate. Consequently, the net energetic cost of synthesizing 1 mol of cysteine under anoxic conditions is only 3 mol of ATP.

10.1128/mBio.01073-20.5 FIG S5 Hydrogen sulfide is a good source of sulfur for E. coli. Log-phase MG1655 (wild-type) cells were washed and diluted at time zero in sulfur-free anoxic growth medium containing 100 μM 18 nonsulfurous amino acids. The medium was supplemented with either 40 μM cystine or 40 μM hydrogen sulfide. Download FIG S5, PDF file, 0.1 MB. Copyright © 2020 Zhou and Imlay. 2020 Zhou and Imlay This content is distributed under the terms of the Creative Commons Attribution 4.0 International license.

10.1128/mBio.01073-20.6 FIG S6 K-12 strain W3110 is unable to assimilate cysteine in the presence of other amino acids. Growth was monitored in anoxic medium in which 40 μM cysteine was provided as the sole sulfur source. The medium contained 100 μM of the 18 nonsulfurous amino acids. Download FIG S6, PDF file, 0.1 MB. Copyright © 2020 Zhou and Imlay. 2020 Zhou and Imlay This content is distributed under the terms of the Creative Commons Attribution 4.0 International license.

### E. coli lacks two known cysteine importers.

These three ATP should further be weighed against the energy that would be required for the synthesis and operation of a notional cysteine importer. An ion-driven importer would in principle expend only one-third of an ATP per cysteine imported and thus would be moderately less expensive than sulfide assimilation. To date, one such importer has been identified, in *Saccharomyces* (52). However, its apparent *K_m_* is remarkably high—55 μM—reflecting weak binding of cysteine. In contrast, the assimilatory amino acid importers of E. coli exhibit *K_m_* values in the nanomolar range for ABC transporters or in the 1 to 5 μM range for secondary transporters (Fig. S1). Because the intestine is packed with competing bacteria, importers must bind trace substrates with high avidity. The yeast-type cysteine importer would seem to be a poor fit for such a habitat. In fact, no homologs are found among the bacteria; the yeast cysteine transporter belongs to a family of import proteins that exist only in fungi (52).

The alternative would be an ABC-type cysteine importer, as transporters of this class exhibit high affinity for their substrates. Indeed, an apparent ABC-type cysteine importer was discovered in Campylobacter jejuni (18). Its periplasmic protein binds cysteine with a *K_D_* of 100 nM, which compares well with the other ABC systems. Such high affinity represents a Δ*G*°′ of binding of approximately 10 kcal; accordingly, the structures of ABC transporters indicates that 2 ATP are hydrolyzed per import of substrate (53). Thus, the operation of such a transporter would consume only 1 fewer ATP than would *de novo* synthesis of cysteine from hydrogen sulfide. Furthermore, a significant fraction of this difference would be expended in the synthesis of the import machinery; in aggregate, we predict that cysteine import would consume 80% as much energy as synthesis (see Text S1C). Thus, under anoxic conditions, it would cost the cell almost as much energy to import cysteine as to synthesize it. We speculate that the modest residual ATP advantage has not imposed enough selective pressure to favor dispersal of the C. jejuni cysteine ABC transporter among the anoxic biota. More broadly, this analysis points out that the economics of biosynthesis are very different under anoxic versus oxic conditions.

Perhaps for this reason, the distribution of this putative cysteine importer is unusually spotty (18). BLAST searches show that an isolate of Bacillus cereus contains a homolog, but most *Bacilli* do not; a homolog is present in Clostridium cellulovorans but not in most *Clostridia*, and so on. This pattern contrasts with the cystine import systems, which are found almost everywhere (2). Coincidentally or not, the discovery and characterization of the cysteine importer occurred in studies of two pathogens that live in reduced oxygen concentrations in their host: Campylobacter jejuni and Neisseria gonorrhoeae (18, 19). Such environments provide an unusual combination of circumstances, in that the low oxygen level permits cysteine to persist without oxidation, but the respiratory metabolism of the bacterium creates a large energetic advantage for cysteine import compared to that for endogenous synthesis.

Finally, we also note that overimport of cysteine might create problems, which might further disfavor the use of cysteine importers. Studies have shown that during the initial import of its oxidized counterpart, cystine, the burgeoning intracellular pool of cysteine disrupts metabolism, because cysteine competes for the active sites of biosynthetic enzymes whose natural substrates are other amino acids (15, 51, 54, 55). Inhibition of threonine deaminase, for example, impairs the synthesis of isoleucine and can cause growth to stall. As noted in the present study, excess cysteine also disrupts iron metabolism and can drive the oxidation of DNA (25). E. coli responds to such a crisis by inducing an amino acid exporter and pumping the excess cysteine back out of the cell (11). Were a dedicated cysteine importer to overshoot as does the cystine importer, the resultant futile cycle would more than exhaust the meager energy advantage that import provides. In contrast, endogenous synthesis from hydrogen sulfide is tightly regulated (56) and does not result in any cysteine excretion. Collectively, these observations suggest that because anaerobic cysteine import might combine high risk with minimal reward, many microbes do without it.

As this paper was being prepared for publication, Oded Lewinson communicated to us that his group had observed binding of cysteine *in vitro* to E. coli TcyJ, the periplasmic binding protein of the ABC-type cystine importer, and that this binding stimulated ATP hydrolysis by the associated TcyLN components (57). Binding at this site is surprising, because the side chain of cysteine (-CH_2_-SH) does not resemble the effective side chain of cystine (-CH_2_-S-S-CH_2_-CH-[COO-]-NH_3_^+^). The modeled binding sites overlapped, although the proposed coordination involved different residues. The binding constant that they observed (∼15 μM) is relatively high and as such is not inconsistent with our data, which showed, for example, a midpoint of CysB deactivation at 20 μM cysteine. However, this *K_D_* seems too high to be effective in the colon, as the reported cysteine concentration is <10 μM (46). More work must be done to inspect the impact of TcyJLN upon cysteine import *in vivo*.

### Amino acid import through noncognate transporters is common but unlikely to be a natural phenomenon.

We observed that the LIV system can import cysteine, but the growth data indicated that this process fails when other amino acids are present. It is worthwhile to demonstrate why this is so. The affinity of the transporter (whether LivK or LivJ is the periplasmic binding protein) is approximately 20-fold higher for Leu, Ile, and Val than for cysteine. This difference turns out to be sufficient to exclude cysteine as a substrate.

Throughout the biota, the leucine, isoleucine, and valine contents of proteomes are approximately 5-fold, 3-fold, and 5-fold higher, respectively, than that of cysteine, and for the sake of this calculation, we presume that similar ratios will pertain in environments in which cell death and degradation have released amino acids. We have measured the maximum flux of Leu into E. coli to be 13 mM/min, whereas log-phase E. coli requires 1.6 mM/min cysteine as a sulfur source (2). Using a standard model whereby the branched-chain amino acids compete with cysteine for import by LIV, we show that natural amino acid ratios would ensure that cysteine import through this system can occur at no more than approximately 0.5% of the transporter *V*_max_, or 70 μM/min (Text S1B). This is only 4% of the cellular sulfur demand. As described in a preceding section, LIV-mediated cysteine import would fail even more completely at the amino acid ratios that are found in the colon. For this reason, the LIV system is inadequate for cysteine acquisition.

The adventitious activity of the LIV system upon cysteine is not incongruous, as LIV has also been shown to operate at low efficiency upon other amino acids, including phenylalanine (42). Presumably, the transporter binds avidly to the carboxylate and amino moieties of all amino acids, while a hydrophobic pocket accommodates neutral side chains with less discrimination. This suits cysteine, whose side chain when protonated associates with hydrophobic environments (30, 31).

Our data reveal other examples of nonspecific amino acid transport. By chance, we observed that the AroP importer is capable of histidine uptake, a phenomenon that others reported previously (39, 40). The affinity of AroP for histidine (*K_m_* = ∼100 μM) is far lower than that of the dedicated HisJQMP histidine importer (*K_m_* = ∼30 nM) (39), but an interesting feature is that at saturating histidine concentrations, AroP can import histidine to a higher internal concentration than HisJQMP. Why would the adventitious transporter outperform the dedicated one in this way? Two facts contribute. First, ion-driven transporters generally exhibit higher flux rates than ABC transporters, since the catalytic cycles of the latter involve protein association/dissociation steps that become rate limiting when substrate concentrations are high. Second, we suspect that ion-driven transporters slow down when the intracellular concentration of their authentic substrates rise high enough to exert product inhibition, which would occur when they bind to the internal aspect of the import channel. However, if the transporter has a relatively low affinity for a pseudosubstrate, its intracellular concentration must rise much higher before inhibition occurs. Thus, when a single amino acid is supplied at high concentration, adventitious transport through a noncognate system may overload the cell. We believe this scenario may explain why, when other amino acids were absent, it was AroP rather than HisJQMP that drove histidine to excessive intracellular concentrations. Similarly, the LIV system enabled cysteine to accumulate to toxic levels; methionine accumulated to Lrp-activating concentrations not by passage through the authentic Met importer, which is saturated by low levels of Met, but only when millimolar levels of methionine drove import through a different system. We regard such transport as a laboratory artifact that is unlikely to occur in natural environments, where bacteria will encounter amino acids in natural mixtures. In that situation, it seems probable that the authentic substrates will outcompete the pseudosubstrates.

## MATERIALS AND METHODS

### Chemicals.

l-Cysteine hydrochloride monohydrate, l-cystine dihydrochloride, and other nonradioactive amino acids were purchased from Sigma-Aldrich. A 30% (wt/wt) solution of hydrogen peroxide (H_2_O_2_), bovine catalase, ovalbumin, *O*-nitrophenol-β-galactoside (ONPG), diethylenetriamine pentaacetic acid (DTPA), dithiothreitol (DTT), 2,2′-dipyridyl, β-mercaptoethanol, antibiotics, and 5-bromo-4-chloro-3-indolyl-β-d-galactopyranoside (X-Gal) were purchased from Sigma-Aldrich. Coomassie reagent was from Thermo Scientific. Medium reagents and buffer chemicals were from Fisher Chemical. Restriction and ligation enzymes were from New England BioLabs. Qiagen kits were used for genomic and plasmid DNA preparation. Colony PCR beads were from GE Healthcare, and PCR reagents were purchased from Invitrogen. DNA sequencing was performed at ACGT, Inc. Radiolabeled l-[1,2,1′,2′-^14^C]cystine and l-[^14^C(U)]leucine were purchased from Perkin Elmer; we note that putative radiolabeled cystine that was received from another supplier was found not to be authentic cystine. Transport measurement filters were purchased from Millipore Sigma (GSWP02500).

### Growth media.

Aerobic growth medium was comprised of minimal A salts (58) supplemented with 0.2% glucose, 0.02% MgSO_4_, and 0.005 mg/ml thiamine. Minimal A salts contain K_2_HPO_4_, KH_2_PO_4_, (NH_4_)_2_SO_4_, and sodium citrate·2H_2_O, adjusted to pH 7.0. For sulfur-free minimal A salts, (NH_4_)_2_SO_4_ was replaced with NH_4_Cl of the same molarity (0.15 M). Unless otherwise specified, aerobic growth media contained no amino acids.

For anaerobic cultures, media were stored in a Coy anaerobic chamber under an atmosphere of 90% nitrogen, 5% hydrogen, and 5% carbon dioxide. Histidine was included in all anaerobic growth media because wild-type MG1655 exhibits anaerobic bradytrophy. Because histidine does not influence cysteine import, figures indicate 0 aa despite its presence.

Sulfur-free medium was prepared by substituting 2.1 mM MgCl_2_ for MgSO_4_. To avoid contaminating sulfur species, all glassware was prerinsed in acid and base and then rinsed at least three times with deionized water before autoclaving. Despite these steps, minimal A salts contain enough contaminating sulfate to enable E. coli growth to an optical density (OD) of ca. 0.1. To remove this sulfur, wild-type cells were grown in the ostensibly sulfate-free medium until growth stopped. These cells were then removed by centrifugation and sterile filtration, thereby rendering sulfate-free medium.

Because of concerns that certain supplements might inadvertently contain sulfate, many experiments were conducted with strains containing deletions in *cysA* and *cysJIH.* These genes encode the primary sulfate importer, sulfite reductase, and PAPS reductase; thus, the mutants are unable to assimilate sulfate or sulfite.

Luria broth (LB; 10 g Bacto tryptone, 5 g yeast extract, 10 g NaCl per liter [58]) was used for strain constructions.

### Growth experiments.

Unless otherwise indicated, cells were grown in minimal glucose medium overnight at 37°C, either in oxic media with full aeration or in anoxic media in the anaerobic chamber, depending upon experimental needs. The overnight cultures were then diluted in the same media to an optical density at 600 nm (OD_600_) of 0.005 and incubated until the cell density reached approximately 0.2, ensuring that they were fully in exponential phase. Cells were then diluted into the same 37°C growth medium to an OD_600_ of 0.0125 to allow the growth behavior to be studied. At this point, select sulfur sources and/or amino acids were provided, depending on the experiment, and the optical densities of the cells were monitored.

The ability of amino acids to supplement various auxotrophs was tested after growing the strains in LB medium to an OD_600_ of approximately 0.7. The cells were collected, washed once in minimal glucose medium, and then diluted into fresh 37°C glucose medium containing 19 amino acids at 200 μM, plus or minus the tested amino acid at 80 μM. Optical density was monitored. By 3 h, supplemented cells had increased in density by at least 10-fold, while unsupplemented cells had grown very little (<2-fold). Strains used were AB1157, AN90-MK3, SSK236, DL39G, JI410, JW3745, and χ478.

### Cysteine stabilization and cystine reduction.

Cysteine is easily oxidized by molecular oxygen. The accordant formation of cystine creates a problem for the study of cysteine import in oxic media. Because trace metals catalyze this reaction (11, 59), short-term experiments were conducted in aerobic medium containing the metal chelator DTPA (1 mM). Longer-term growth experiments were performed in the anaerobic chamber.

Reduced radiolabeled cysteine was prepared by reducing [^14^C]cystine (100 μM) with freshly prepared dithiothreitol (DTT; 5 mM). This reduction process was performed at 37°C for at least 30 min under oxic conditions in minimal A medium, and 1 mM DTPA was supplemented. Reduction of the cystine was confirmed by thin-layer chromatography (TLC). Briefly, 4.5 μl cystine (63 mM) and the cystine-DTT (5 mM cystine, 10 mM DTT, 1 mM DTPA in minimal A buffer) reaction products were loaded onto TLC plates. The plates were carefully angled into a 12:3:5 mixture of 1-butanol, glacial acetic acid, and deionized H_2_O, and the chromatography was performed for approximately 1 h, until the buffer front had diffused two-thirds of the height of plate. The plates were dried in a fume hood, and film was affixed to the TLC plate and stored in a cassette at −80°C for more than 24 h. Reduced cysteine migrated to the upper portion of the plate, while cystine remained near the bottom of the plate.

### Strain construction and plasmid purification.

Null mutations in *aroP*, *liv*(*KMGHF*), and *metN* were generated by the phage λ Red recombination method (60). The targeted open reading frame was replaced by the kanamycin cassette from pKD4 or the chloramphenicol cassette from pKD3, with FLT sites flanking the drug marker on both sides. The FLT-flanked antibiotic resistant markers were eliminated by the FLP activity of pCP20, and the strains were then cured of pCP20 by incubation at the nonpermissive temperature (42°C). This procedure generates nonpolar mutations. Other mutations were transferred to working strains by P1 transduction (58) from mutants that were obtained from the E. coli Genetic Stock Center. All mutations were confirmed by PCR analysis.

A single-copy *yhaO-lacZ* transcriptional fusion (61) was created by amplifying the promoter region, consisting of approximately 500 bp upstream of *yhaO* through its ATG (+1) site. This target region was then ligated to the *lacZ* coding region in pSJ501. The resultant *yhaO-lacZ* fusion was integrated into the genome at the lambda attachment site with the help of pSJ130, which includes the lambda integration genes. The final construct was confirmed by PCR and DNA sequencing.

The plasmids pBR322, pCysB*, and pCP20 were purified by the Qiagen kit from strains KCI1234, KCI1232, and Lem35, respectively.

### Tests of H_2_O_2_ sensitivity.

Cells were diluted from overnight cultures to an OD_600_ of 0.005 in 37°C oxic minimal glucose medium; they were then cultured for four generations to ensure that they were fully in exponential phase. Once cultures had reached an OD_600_ of ∼0.1, they were diluted in the same 37°C medium to an OD_600_ of 0.025. DTPA (1 mM) was supplied to prevent cysteine oxidation. Cysteine and/or amino acids were added to the culture for 3 min, followed by 2.5 mM H_2_O_2_. After 4 min, catalase (130 U/ml, final concentration) was added to the cultures to remove H_2_O_2_, and aliquots of cells were diluted in LB and plated on LB plates using 0.8% top agar. Colonies were counted the next day. An aliquot of cells was also plated before H_2_O_2_ addition, enabling the calculation of percent survival.

### Analysis of gene expression.

To avoid cysteine oxidation, expression of the *yhaO-lacZ* fusion was determined under anoxic conditions. Cultures (25 ml) were inoculated from overnight samples to an OD_600_ of 0.003 in minimal glucose medium, with or without 18 amino acids (20 μM each). Cells were grown to an OD_600_ of 0.020. Different concentrations of cysteine were added, and cultivation continued to an OD of ∼0.050, when cells were harvested.

To test the induction of the *tcyP*-*lacZ* fusion, cultures (25 ml) were inoculated from overnight samples to an OD_600_ of 0.003 in anoxic minimal glucose medium with or without 18 amino acids (20 μM) and with or without different concentrations of cysteine or cystine. Cells were cultured for four generations to an OD_600_ of 0.050 prior to harvesting.

In the experiments reported in Fig. 7A and B and 8C, cysteine (200 μM) and methionine (600 μM or 5 mM) were added when cells were diluted from an overnight culture to an OD_600_ of 0.0125 in minimal glucose medium, and cells were harvested at an OD_600_ of 0.2. The expression of *lacZ* fusions was evaluated by β-galactosidase assays (62). Twenty milliliters of cell cultures was washed with ice-cold 50 mM Tris-HCl (pH 8.0), resuspended in 2 ml of the same buffer, and lysed by French press. The cell lysates were further centrifuged (17,000 × g) to remove debris prior to the assay. Absorbance was monitored at 420 nm for 10 min. One unit represents 1 nmol of ONPG cleaved per min. Total protein was measured using the Coomassie protein assay (Thermo Scientific), with ovalbumin as the protein standard.

In some experiments involving the expression of gene fusions (Fig. 5A and B and 7C), cysteine was provided to cells at low micromolar concentrations. Our lab determined that E. coli requires 64 μM of sulfur atoms to grow to an OD_600_ of 1.0 (2). Due to the risk that cells might exhaust the low concentrations of cysteine, in these experiments, cells were cultured only to an OD_600_ of 0.040 to 0.050. Carrier cells (SJ130, Δ*lacZ*) were then added during harvest to increase the total harvest OD_600_ to 0.10 to help centrifuge the cells into a firm pellet. The measured activity was corrected for the efficiency of cell lysis by normalizing to the amount of protein divided by the amount of carrier cells (OD × volume) added to the sample.

### RNA sequencing analysis.

Three biological replicates of MG1655 were grown for more than six generations to an OD_600_ of 0.1 in aerobic minimal glucose medium supplemented with 0.5 mM histidine and aromatic amino acids. The culture was divided in two; one subculture was supplemented with 50 μM cystine, the other one was not. After 10 min of further incubation, three replicate pairs were harvested for RNA and sequenced, as described previously (63).

### Measurements of cysteine and leucine import.

Overnight cultures in oxic minimal glucose medium were diluted to an OD_600_ of 0.013 and grown for four generations until an OD_600_ of 0.2 was reached. Cells were centrifuged and resuspended in equal volumes of the same medium. Cells were incubated for 5 min with 80 μg/ml chloramphenicol to block protein synthesis. l-[1,2,1′,2′-^14^C]Cystine (e.g., 100 μM) was freshly reduced to cysteine by DTT, and DTPA (1 mM) was supplied to the cocktail mix to suppress cysteine oxidation. The final [^14^C]cysteine cocktail in sulfate medium was added to cells at 37°C to establish a final concentration of 20 μM and, depending upon the experiment, 3 to 40 mCi/mmol cysteine. The cocktail mix (70 μl) was then aliquoted into 630 μl of prewarmed chloramphenicol-treated cells. At intervals, the new mixture (cocktail plus cells) was aliquoted (70 μl) onto a filter on a filtration manifold and washed three times with 2.5 ml of wash buffer (100 mM Tris, 0.15 M NaCl, 0.5 mM MgCl_2_ at room temperature). Wash times were ca. 5 s; the delay applied to all time points and was remedied by using the slope of the time course. Filters were dried by a heat lamp, transferred to vials containing 5 ml scintillation fluid, and analyzed by scintillation counting. In some experiments, other unlabeled amino acids were added to cells prior to the addition of cysteine so that their ability to inhibit cysteine import could be appraised. l-[^14^C(U)]Leucine import was measured according to the same protocol as for cysteine. The intracellular concentrations of cysteine and leucine were calculated using the fact that 1 ml of E. coli at an OD_600_ of 1 contains 0.5 μl of cytoplasm (64). Therefore, from the specific activities of the radiolabeled amino acids and the amount of cell-associated radioactivity, the cytoplasmic concentrations of the imported amino acids were derived, and the import rates are presented as millimolar cytoplasmic cysteine per unit time.

10.1128/mBio.01073-20.9 TABLE S2 Strains used in this study. Download Table S2, DOCX file, 0.1 MB. Copyright © 2020 Zhou and Imlay. 2020 Zhou and Imlay This content is distributed under the terms of the Creative Commons Attribution 4.0 International license.

10.1128/mBio.01073-20.10 TABLE S3 PCR primers. Download Table S3, DOCX file, 0.1 MB. Copyright © 2020 Zhou and Imlay. 2020 Zhou and Imlay This content is distributed under the terms of the Creative Commons Attribution 4.0 International license.
