# Supplementary figures and images for: Escherichia coli K-12 Lacks a High-Affinity Assimilatory Cysteine Importer
Source: mBio. 2020 Jun 9;11(3):e01073-20. doi: 10.1128/mBio.01073-20 (PMC7373191; doi:10.1128/mBio.01073-20)

A. ABC-type transporters.

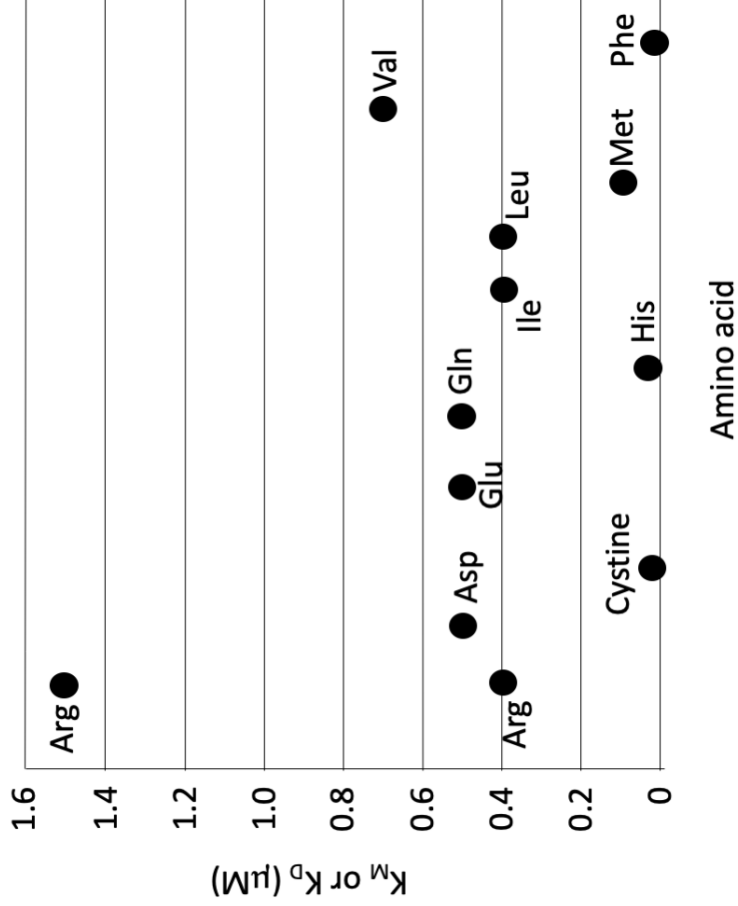

B. Ion-driven transporters.

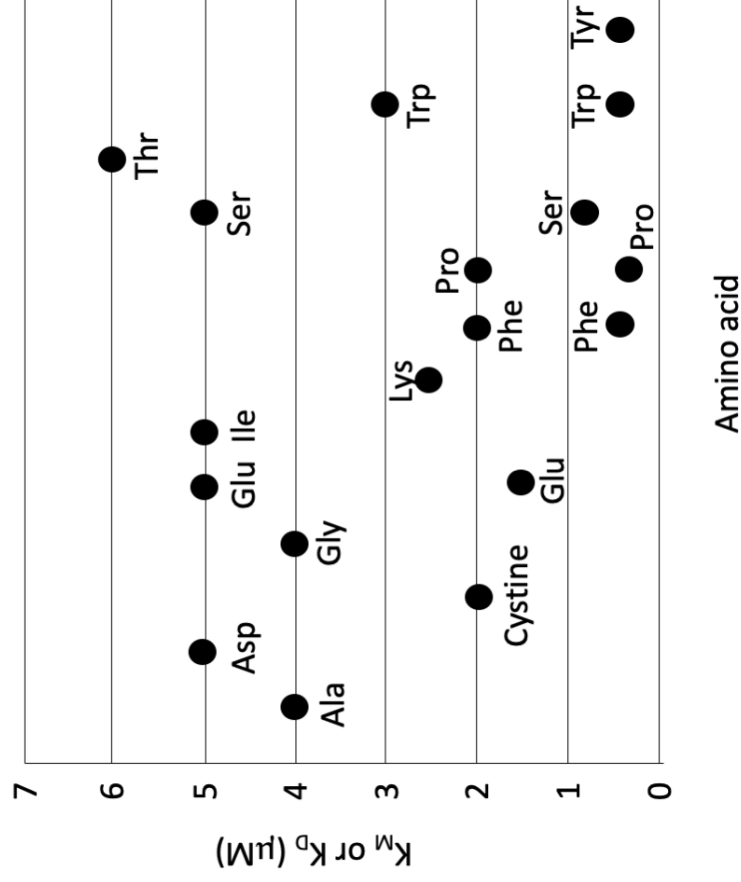

Supplement: FIG S1 [file mBio.01073-20-sf001.pdf]

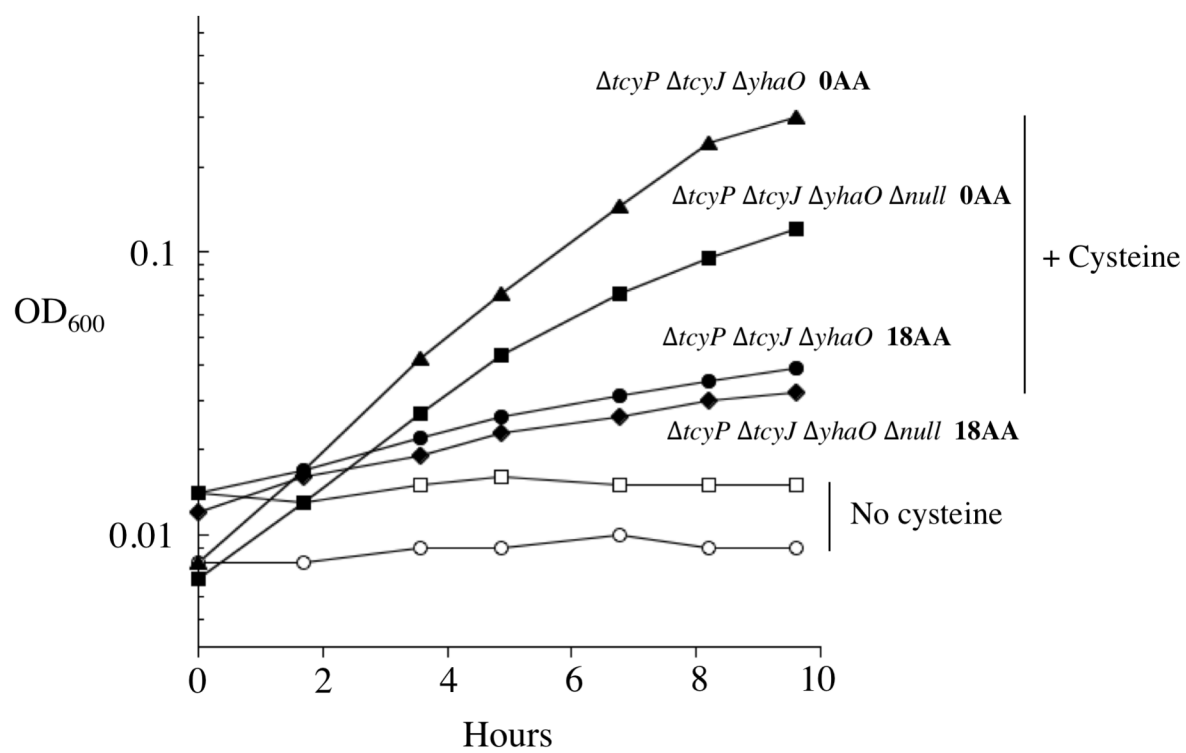

Supplement: FIG S2 [file mBio.01073-20-sf002.pdf]

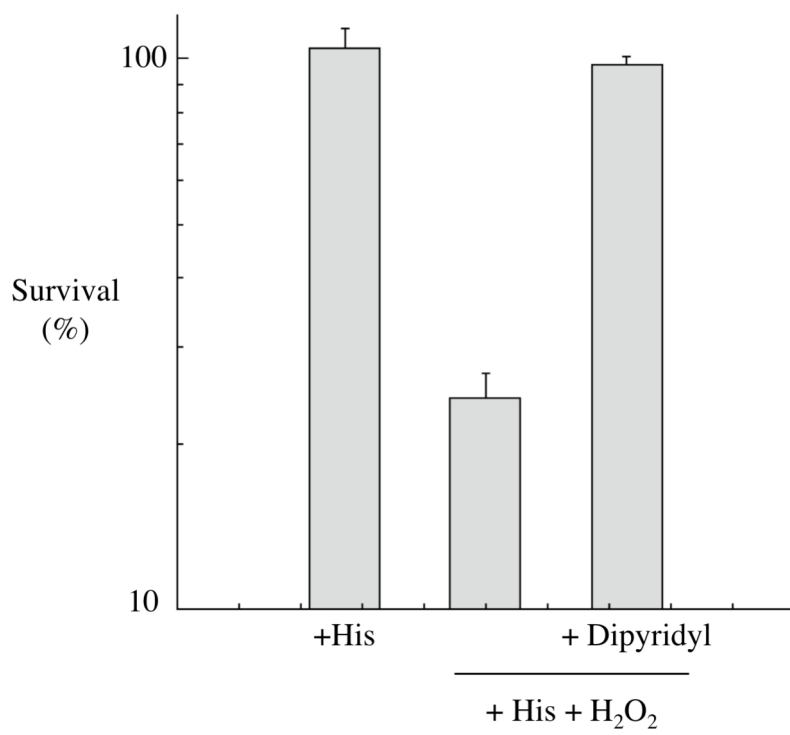

Supplement: FIG S3 [file mBio.01073-20-sf003.pdf]

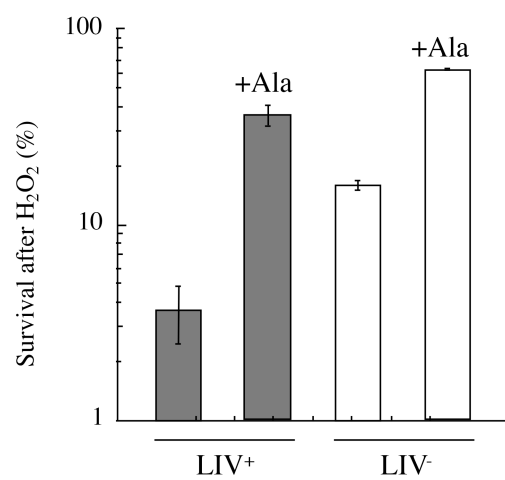

Supplement: FIG S4 [file mBio.01073-20-sf004.pdf]

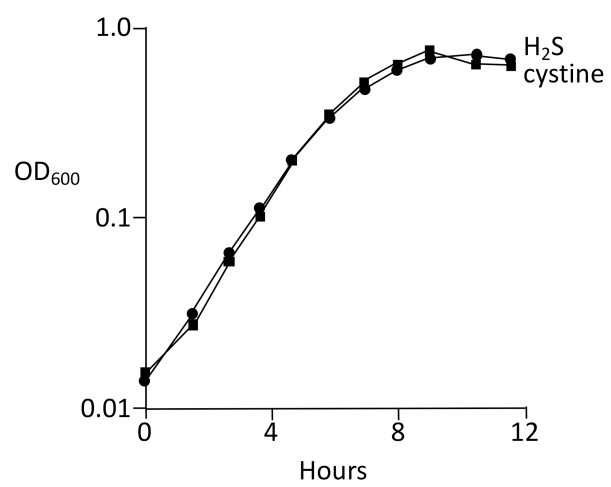

Supplement: FIG S5 [file mBio.01073-20-sf005.pdf]

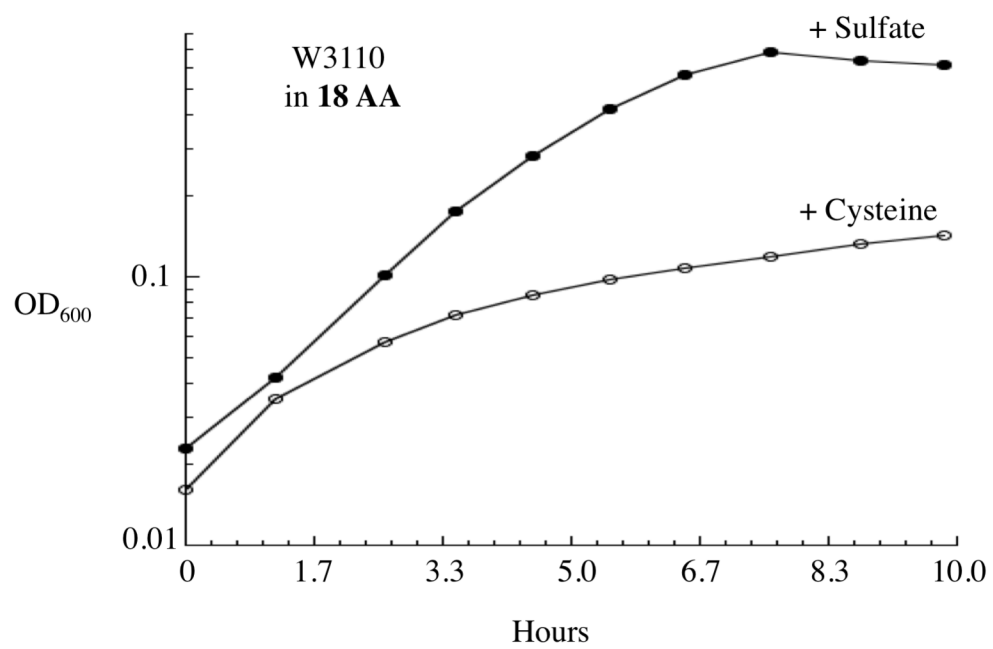

Supplement: FIG S6 [file mBio.01073-20-sf006.pdf]
