# Supplementary material for: Escherichia coli K-12 Lacks a High-Affinity Assimilatory Cysteine Importer
Source: mBio. 2020 Jun 9;11(3):e01073-20. doi: 10.1128/mBio.01073-20 (PMC7373191; doi:10.1128/mBio.01073-20)
Supplement: TABLE S1 [file mBio.01073-20-st001.docx]

**Table S1. Known high-affinity importers of L-amino acids in *E. coli*.** Compiled information is most accessible through EcoCyc (ecocyc.org).

| **Amino acid** | **ATP-type** | **Ion-driven** |
| --- | --- | --- |
| Alanine |  | YaaJ, CycA |
| Arginine | ArgT-HisQPM, ArtJIMQP | ArcD |
| Asparagine |  | AnsP |
| Aspartic acid | GltIKJL | DcuB, GltP |
| Cystine | TcyJLN | TcyP |
| Glycine |  | YaaJ, CycA |
| Glutamine | GlnQPH |  |
| Glutamic acid | GltIKJL | GltP, GltS, GadC |
| Histidine | HisJQPM |  |
| Isoleucine | LivFGHMKJ | BrnQ |
| Leucine | LivFGHMKJ | BrnQ |
| Lysine | ArgT-HisQPM | LysP, CadB |
| Methionine | MetINQ |  |
| Phenylalanine | LivFGHMK | PheP, AroP |
| Proline |  | PutP, ProP |
| Serine |  | TdcC, SdaC, SstT |
| Threonine |  | TdcC, SstT |
| Tryptophan |  | Mtr, AroP |
| Tyrosine |  | TyrP, AroP |
| Valine | LivFGHMKJ | BrnQ |
| Cysteine |  |  |
