# Supplementary material for: Escherichia coli K-12 Lacks a High-Affinity Assimilatory Cysteine Importer
Source: mBio. 2020 Jun 9;11(3):e01073-20. doi: 10.1128/mBio.01073-20 (PMC7373191; doi:10.1128/mBio.01073-20)
Supplement: TEXT S1 [file mBio.01073-20-s0001.docx]

**Supplemental Text.**

**(A) Calculation of the energetic cost of cysteine synthesis.**

*The cost of cysteine synthesis under fermentative conditions is 3 ATP.*

In the anoxic gut the predominant catabolic action of *E. coli* is likely to be the fermentation of carbohydrates. The fermentation of 1 mole of glucose proceeds through glycolysis to 2 moles of pyruvate, yielding 2 moles of ATP. Pyruvate is split to 2 moles of acetyl-CoA (plus 2 formate). During glycolysis 2 moles of NADH are generated; the redox balance is restored when they are consumed during conversion of one acetyl-CoA to ethanol. The second mole of acetyl-CoA is converted to acetate, with formation of one more mole of ATP. Therefore, the net yield is 3 ATP per glucose consumed.

Cysteine biosynthesis diverts one molecule of the glycolytic intermediate 3-phosphoglycerate, and so the energetic cost of the process must consider both the energy expended in the cysteine biosynthetic pathway itself and the energy that is lost from the failure to normally process the 3-phosphoglycerate. The biosynthetic arm of this process is:

3PG + NAD^+^ + AcCoA + H_2_S + NADPH + NH_4_^+^ 🡪 cysteine + NADH + NADP^+^ + Pi + CoA + acetate

where 3PG is 3-phosphoglycerate, AcCoA and CoA represent acetyl-coenzyme A and coenzyme A, and Pi is orthophosphate. Notably, the pathway is redox-balanced. The net electron transfer from NADPH to NADH is addressed below.

Cysteine biosynthesis can be balanced with glucose fermentation:

2 glucose + NADPH + NAD^+^ + NH_4_^+^ + H_2_S 🡪 cysteine + NADH + NADP^+^ + 2 ethanol + 3 formate

In this course of cysteine synthesis, three moles of ATP are produced per 2 moles of glucose consumed. Were cysteine instead obtained from the medium, consumption of the same two moles of glucose would generate six moles of ATP. Thus the apparent expense is 3 moles of ATP per mole of cysteine synthesized.

In principle the net transfer of electrons from NADPH to NAD^+^ may cost additional energy, since the reverse reaction mediated by transhydrogenase is coupled to proton conduction across the membrane—the equivalent of ~ 1/3 ATP (1, 2). If nitrogen is assimilated from ammonia by glutamine synthetase rather than glutamate dehydrogenase, another ATP would be expended; were other nitrogen sources used, this would not be the case. Thus our estimate of ~3 ATP equivalents expended per cysteine synthesized comes with some uncertainty.

*The cost of cysteine synthesis under respiratory conditions may be up to 32 ATP.*

In contrast, the effective cost of cysteine synthesis is 10-fold higher under aerobic conditions. In oxic habitats the prevailing inorganic sulfur species will likely be sulfate rather than hydrogen sulfide. The diversion of 3-phosphoglycerate to biosynthesis comes at the cost of the 2 ATP equivalents and five electron pairs, as these would otherwise be produced by substrate-level phosphorylation and oxidation of the C3 molecule to CO_2_. Four electron pairs, when introduced into the respiratory chain at the level of NADH, each drive the translocation of 8 protons—which are conserved as ~2 2/3 ATP; a fifth electron pair, at the level of FADH_2_, generates 1 1/3 ATP. Thus the net expense of 3-phosphoglycerate diversion, in place of 3-phosphoglycerate degradation, is approximately 14 ATP equivalents. (This value would be lower if respiration were mediated by non-translocating NADH dehydrogenase II and/or cytochrome bd oxidase; however, under the energy-poor situation for which cell-economy calculations are most relevant, the electron flux is presumably routed through the proton-translocating NADH I dehydrogenase and cytochrome bo oxidase. These calculations also presume an H^+^/ATP coupling number of 3 for the ATPase, as determined by Kashket (2); it has been proposed that this value may vary somewhat with growth conditions (3). Neither uncertainty will impact the overall conclusion of this analysis.)

Sulfate reduction to sulfide consumes four NAD(P)H electron pairs and two ATP equivalents; two additional ATP equivalents are expended for sulfate import, and two are lost via AcCoA deacetylation. Five electron pairs must be transferred from NADH to NADP^+^ (including that for NH_4_^+^ assimilation) adding a net transhydrogenation cost of 1 2/3 ATP. Thus, the biosynthetic pathway per se expends 18 ATP. The overall effective cost, then, of aerobic cysteine biosynthesis is 32 ATP.

When cystine serves as an aerobic cysteine source, one mole of NADPH is employed to reduce it to two moles of cysteine. The effective cost is 1/3 to 2 ATP for transport, depending upon whether TcyP or TcyJLN is the importer, and a cost of ~ 3 ATP to provide NADPH. Since 2 moles of cysteine are recovered, the net expense is about 2 moles ATP per mole of cysteine. Thus, in oxic environments cystine is a far more economical cysteine source than is de novo synthesis. The take-home message: cystine import provides great energy savings under aerobic conditions, but cysteine import provides almost no savings under anaerobic conditions.

Analogous calculations can be made for the use of sulfate (2 2/3 ATP) and cystine (-1/6 ATP) under anoxic conditions, and for the acquisition of cysteine from sulfide assimilation (16 1/3 ATP) and cysteine import (2 ATP) under oxic conditions. Cystine use under fermentative conditions actually yields a small amount of ATP, as its action as an electron sink enables more acetyl-CoA to be converted to acetate (rather than reduced to ethanol), with concomitant ATP production. The predicted ATP expenditures with various sulfur sources are presented in Table 3. Note, however, that chemical oxidation by molecular oxygen is likely to minimize the presence of hydrogen sulfide or cysteine in oxic environments, and chemical reduction by ambient hydrogen sulfide may minimize cystine availability in the gut.

**(B) Calculation of the potential for the LIV system to import cysteine.**

The LivJ periplasmic binding protein exhibits high affinities for leucine (K_D_ = 400 nM), isoleucine (400 nM), and valine (700 nM) (4). Our competition experiments suggest that the affinity for cysteine (half-maximum rate at 20 μM) is about 20-fold lower than that for leucine; thus we estimate the apparent K_M_ values to be approximately 20 μM Cys, 1 μM Leu, 1 μM Ile, and 1.8 μM Val. The relative abundances of these amino acids in the natural proteome is approximately 1 Cys: 5 Leu: 3 Ile: 5 Val (5). These values can be placed into the standard equation for kinetic competition:

(1) V = V_max_ [S]{αK_M_ + [S]}^-1^, where α = 1 + [I]/K_I_

where V is the rate of substrate turnover, [S] and [I] represent the concentrations of substrate and inhibitor, and K_M_ and K_I_ represent binding constants of substrate and inhibitor. The denominator of the equation can be expanded to accommodate multiple inhibitors with a term [I]/K_I_ for each.

(2) V = V_max_ [S]{K_M_(1 + I_1_/K_I1_ + I_2_/K_I2 +_ I_3_/K_I3_) + [S]}^-1^

By inserting the above ratios of substrate (cysteine) and inhibitor (leucine, isoleucine, valine) concentrations and binding constants, one obtains:

(3) V = V_max_ [Cys](20 μM + 218[Cys])^-1^

(4) At saturating [Cys], V = 0.0046 V_max_

Since we measured the V_max_ of leucine import to be 13 mM/min, V cannot exceed 0.060 mM/min. The cellular demand for sulfur atoms under these conditions is 1.6 mM/min; thus, the maximum rate of cysteine transport through the LIV system is 4% of the cellular demand. Calculations show that environmental Leu, Ile, and Val concentrations must be depleted by 96% relative to cysteine before the competition is relieved enough that cysteine import could match the cellular demand. For this reason, the LIV system cannot be regarded as an adequate source of cysteine in any environment in which the mixture of amino acids resembles their relative abundances in the proteome. This conclusion would be more emphatic were the calculation performed using the natural abundances of amino acids in the colon. In that environment they are further tilted towards branched-chain amino acids and alanine, while cysteine was not detected (< 10 μM) (5a).

**(C) Calculation of the energetic cost of synthesizing an ATP-dependent cysteine importer.**

The energy expended per amino acid synthesized was calculated for all twenty amino acids, using the conditions of glucose fermentation, as described in Supplement S1 for cysteine. As noted, some ambiguity arises when considering different routes of NADP^+^ reduction, but the approach will certainly suffice to give an approximate answer. In general, a pathway that consumes NAD(P)H will improve the ATP yield by 0.5 mole ATP, as it spares acetyl-CoA from conversion to ethanol (for the purpose of redox-balancing) and instead directs it towards acetate formation, which generates ATP.

The net biosynthetic ATP expenses for amino acids were calculated for a glucose fermentation: alanine, 0.5 ATP; arginine, 9 ATP; asparagine, 4.5 ATP; aspartate, 1.5 ATP; serine, 2 ATP; cysteine, 3 ATP; glutamate, 3 ATP; glutamine, 4 ATP; glycine, 2 ATP; histidine, 8 ATP; threonine, 2.5 ATP; isoleucine, 2.5 ATP; valine, 1 ATP; leucine, 2.5 ATP; lysine, 3 ATP; methionine, 4.5 ATP; phenylalanine, 7 ATP; proline, 3 ATP; tryptophan, 10.5 ATP; tyrosine, 7.5 ATP.

The amino acid composition was determined for the *Helicobacter pylori* homolog of the apparent ABC system for cysteine transport (6). The total biosynthetic energy cost was calculated by multiplying the amino acid content by the ATP devoted to their syntheses, and adding 4 ATP equivalents for each peptide bond. We thereby estimated the ATP demand for synthesis of the cysteine binding protein (annotated in the NCBI database as GlnH) to be 1975 equivalents; for the GlnQ ATPase subunit, 1828; for the GlpP1 permease subunit, 1625; and for the second, GlnP2 permease subunit, 1598. When *E. coli* is cultivated in minimal medium, the periplasmic binding proteins of its ABC transporters are approximately 30-fold more abundant than those of the membrane complex (7). Therefore, the total ATP cost of the membrane complex, coupled to 30 copies of the periplasmic binding protein, was about 66,000 ATP equivalents. In aerobic sulfate medium the *E. coli* cell contains roughly 400 copies of the membrane components of its TcyJLN cystine-import system; if the notional GlnH_30_-Q_2_-P1-P2 system were equally abundant, at a doubling time of 60 min (k = 0.0116 min^-1^), the rate of transporter synthesis was therefore about 4.6 min^-1^ at a cost of 304,000 ATP min^-1^.

In this medium *E. coli* requires a flux of 830,000 cysteine per minute to fulfill its sulfur demand. If this demand were met by cysteine import, at a cost of 2 ATP per molecule imported, then total ATP expenditure for import would be 304,000 + (2 x 830,000) = 1,960,000 ATP min^-1^. Therefore, synthesis of the import machinery is a relatively minor element (15%) of the energy cost. To meet the same demand by endogenous cysteine synthesis would cost 2,490,000 ATP. This estimate suggests that cysteine import would be only marginally more economical—20%—than synthesis.

**References for Supplementary Material.**

1. Zhang Q, Padayati PS, Leung JH. 2017. Proton-translocating nicotinamide nucleotide transhydrogenase: a structural perspective. Front Physiol 8:1-6.

2. Kashket ER. 1982. Stoichiometry of the H^+^-ATPase of growing and resting, aerobic *Escherichia coli*. Biochemistry 21:5534-5538.

3. Tomashek JJ, Brusilow WSA. 2000. Stoichiometry of energy coupling by proton-translocating ATPases: a history of variability. J Bioenerg Biomembr 32:493-500.

4. Adams MD, Maguire DJ, Oxender DL. 1991. Altering the binding activity and specificity of the leucine binding proteins of *Escherichia coli*. J Biol Chem 266:6209-6214.

5. Neidhardt FC, Umbarger HE. 1996. Chemical composition of *Escherichia coli*. In: *Escherichia coli* and *Salmonella* Edited by FC Neidhardt *et al* ASM Press, Washington, DC.

5a. Smith, EA, Macfarlane, GT. 1998. Enumeration of amino acid fermenting bacteria in the human large intestine: effects of pH and starch on peptide metabolism and dissimilation of amino acids. FEMS Micro Ecol 25:355-368.

6. Muller A, Thomas GH, Horler R, Brannigan JA, Blagova E, Levdikov VM, Fogg JJ, Wilson KS, Wilkinson AJ. 2005. An ATP-binding cassette-type cysteine transporter in *Campylobacter jejuni* inferred from the structure of an extracytoplasmic solute receptor protein. Mol Microbiol 57:143-155.

7. Li G-W, Burkhardt D, Gross C, Weissman JS. 2014. Quantifying absolute protein synthesis rates reveals principles underlying allocation of cellular resources. Cell 157:624-635.

8. Datsenko KA, Wanner BL. 2000. One-step inactivation of chromosomal genes in *Escherichia coli* K-12 using PCR products. Proc Natl Acad Sci U S A 97:6640-5.

9. Colyer TE, Kredich NM. 1994. Residue threonine-149 of the *Salmonella typhimurium* CysB transcription activator: mutations causing constitutive expression of positively regulated genes of the cysteine regulon. Mol Microbiol 13:797-805.

10. Imlay KRC, Korshunov S, Imlay JA. 2015. The physiological roles and adverse effects of the two cystine importers of *Escherichia coli*. J Bacteriol 197:3629-3644.

11. Cherepanov PP, Wackernagel W. 1995. Gene disruption in *Escherichia coli*: TcR and KmR cassettes with the option of Flp-catalyzed excision of the antibiotic-resistance determinant. Gene 158:9-14.

12. Neidhardt FC, R Curtiss I, Ingraham JL, Lin ECC, Low KB, Magasanik B, Reznikoff WS, Riley M, Schaechter M, Umbarger HE. 1996. *Escherichia coli* and *Salmonella*. ASM Press, Washington, D.C.
