# Supplementary material for: Escherichia coli K-12 Lacks a High-Affinity Assimilatory Cysteine Importer
Source: mBio. 2020 Jun 9;11(3):e01073-20. doi: 10.1128/mBio.01073-20 (PMC7373191; doi:10.1128/mBio.01073-20)
Supplement: TABLE S2 [file mBio.01073-20-st002.docx]

**Table S2. Strains used in this study.**

| Strain name | Genotype | Source |
| --- | --- | --- |
| AB1157 | *thr-1 leuB6 proA2 his-4 thi-1 argE2 lacY1 glaK2 rpsL supE44 ara-14 xyl-15 mtl-1 tsx-33* | Lab collection |
| AN92-MK3 | *entA::kan proA2 argE3 pheA1 tyrA4 trp-401 aroB351 rpsL* | Lab collection |
| BW25113 | *lacI*^q^*rrnB*_T14_ Δ*lacZ*_WJ16_*hsdR514* Δ*araBAD*_AH33_ Δ*rhaBAD*_LD78_  pKD46-TS | (8) |
| DL39G | *aspC13 fnr-25 glyA42::*Tn*5 ilvE2 tyrB507* | Lab collection |
| JI410 | *gltA9 gal76::*Tn*10* | Lab collection |
| JW3745 | Δ*(araD-araB)567* Δ*lacZ4787(::rrnB-3) ilvA::kan rph-1* Δ *(rhaD-rhaB)568 hsdR514* | Lab collection |
| KCI826 | Δ*cysJIH1* Δ*cysA751::*Km | Lab collection |
| KCI1205 | Δ*lacZ1* attλ[pSJ501::*tcyP’-lacZ*^+^]~*cat* | Lab collection |
| KCI1232 | Δ*lacZ1* attλ[pSJ501::*tcyP’-lacZ*^+^] with pCysB* | (9, 10) |
| KCI1234 | Δ*lacZ1* attλ[pSJ501::*tcyP’-lacZ*^+^] with pBR322 | (10) |
| Lem35 | Contains pCP20 | Lab collection (11) |
| MG1655 | F^-^ wild-type *E. coli* | (12) |
| SJ130 | Δ*lacZ1* | Lab collection |
| SSK236 | As MG1655 plus *metC::*Tn*10* | Lab collection |
| SSK250 | Δ(*alaE::*Km)1*::*Φ(*alaE’-‘lacZ lacY*^+^)1~Km Δ*lacZ*1 | Lab collection |
| W3110 | IN*(rrnD-rrnE)1 rph-1* | Lab collection |
| χ478 | *ara-14 leuB6 azi-6 tonA23 lacZ36 proC32 tsx-67 purE42 supE44 trpE38 lysA23 rpsL109 xyl-5 mtl-1 metE70 thi-1* | Lab collection |
| ZYD15 | Δ*tcyP1* Δ*tcyJ1* Δ*yhaO728* | This work |
| ZYD19 | Δ*tcyP1* Δ*tcyJ1* Δ*yhaO728* Δ*hisJ730::*Km | This work |
| ZYD30 | Δ*tcyP1* Δ*tcyJ1* Δ*yhaO728* Δ*aroP730::*Km | This work |
| ZYD34 | Δ*tcyP1* Δ*tcyJ1* Δ*yhaO728* Δ*hisJ730* Δ*aroP730::*Km | This work |
| ZYD77 | Δ*tcyP1* Δ*tcyJ1* Δ*yhaO728* Δ*yaaJ727::*Km | This work |
| ZYD79 | Δ*tcyP1* Δ*tcyJ1* Δ*yhaO728* Δ*livF784::*Km | This work |
| ZYD81 | Δ*tcyP1* Δ*tcyJ1* Δ*yhaO728* Δ*cycA757::*Km | This work |
| ZYD83 | Δ*tcyP1* Δ*tcyJ1* Δ*yhaO728* Δ*brnQ765::*Km | This work |
| ZYD107 | Δ*tcyP1* Δ*tcyJ1* Δ*yhaO728* Δ*liv(KMGHF)1::cat* Δ*brnQ765* | This work |
| ZYD109 | Δ*tcyP1* Δ*tcyJ1* Δ*cysA751::kan* Δ*cysJIH1::cat* | This work |
| ZYD114 | Δ*tcyP1* Δ*tcyJ1* Δ*yhaO728* Δ*cysA751::*Km Δ*cysJIH1::cat* | This work |
| ZYD119 | Δ*tcyP1* Δ*tcyJ1* Δ*yhaO728* Δ*brnQ765* Δ*liv(KMGHF)1::cat* Δ*cycA757::*Km | This work |
| ZYD128 | *fruR11::*Tn*10*~*leuA781::*Km Δ*tcyP1* Δ*tcyJ1* Δ*yhaO728* Δ*brnQ765* Δ*liv(KMGHF)1::cat* | This work |
| ZYD134 | Δ*tcyP1* Δ*tcyJ1* Δ*yhaO728* Δ*brnQ765* Δ*liv(KMGHF)1::cat* Δ*yaaJ727::*Km | This work |
| ZYD138 | Δ*tcyP1* Δ*tcyJ1* Δ*yhaO728* Δ*brnQ765* Δ*liv(KMGHF)1* Δ*yaaJ727* Δ*cycA757::*Km | This work |
| ZYD141 | Δ*tcyP1* Δ*tcyJ1* Δ*yhaO728* Δ*liv(KMGHF)1::cat* Δ*cycA757::*Km | This work |
| ZYD143 | Δ*tcyP1* Δ*tcyJ1* Δ*yhaO728* Δ*cycA757::*Km Δ*brnQ765* | This work |
| ZYD154 | Δ*tcyP1* Δ*tcyJ1* Δ*yhaO728* Δ*lacZ1::cam* attλ[pSJ501::*yhaO’-lacZ*^+^] | This work |
| ZYD156 | Δ*tcyP1* Δ*tcyJ1* Δ*lacZ1::cat* attλ[pSJ501::*yhaO’-lacZ*^+^] | This work |
| ZYD162 | Δ*tcyP1* Δ*tcyJ1* Δ*lacZ1::cat* attλ[pSJ501::*yhaO’-lacZ*^+^] with pBR322 | This work |
| ZYD164 | Δ*tcyP1* Δ*tcyJ1* Δ*lacZ1::cat* attλ[pSJ501::*yhaO’-lacZ*^+^] with pCysB* | This work |
| ZYD176 | Δ*tcyP1* Δ*tcyJ1* Δ*yhaO728* Δ*yaaJ727* Δ*cycA757::*Km | This work |
| ZYD180 | Δ*tcyP1* Δ*tcyJ1* Δ*yhaO728* Δ*yaaJ727* Δ*cycA757::*Km Δ*liv(KMGHF)1::cat* | This work |
| ZYD184 | Δ*tcyP1* Δ*tcyJ1*Δ*yhaO728* Δ*yaaJ727* Δ*liv(KMGHF)1::cat* | This work |
| ZYD186 | Δ*tcyP1* Δ*tcyJ1* Δ*yhaO728* Δ*brnQ765* Δ*yaaJ727::*Km | This work |
| ZYD210 | Δ*tcyP1* Δ*tcyJ1* Δ*yhaO728* Δ*brnQ765* Δ*liv(KMGHF)1* Δ*yaaJ727* Δ*cycA757* Δ*cysA751::*Km Δ*cysJIH1::cat* | This work |
| ZYD214 | Δ*tcyP1* Δ*tcyJ1* Δ*yhaO728* Δ*cycA757* Δ*brnQ765* Δ*yaaJ727::*Km | This work |
| ZYD221 | Δ*tcyP1* Δ*tcyJ1* Δ*yhaO728* Δ*lacZ1* attλ[pSJ501::*tcyP’-lacZ*^+^] | This work |
| ZYD239 | Δ*metN1::cat* Δ(*alaE::*Km)1*::*Φ(*alaE’-‘lacZ lacY*^+^)1~Km Δ*lacZ*1 | This work |
