# Supplementary material for: Escherichia coli K-12 Lacks a High-Affinity Assimilatory Cysteine Importer
Source: mBio. 2020 Jun 9;11(3):e01073-20. doi: 10.1128/mBio.01073-20 (PMC7373191; doi:10.1128/mBio.01073-20)
Supplement: TABLE S3 [file mBio.01073-20-st003.docx]

**Table S3. PCR primers.**

| Purpose | Sequence | Relevant strains and plasmid |
| --- | --- | --- |
| *aroP* deletion | F: 5’-CACGCATCACTGCGTAG  ATCAAAAAAACAACCACCGCACGAGGTTTCATGTGTAGGCTGGAGCTGCTTCG  R: 5’-CACCCTGTACGGGTG  AGGGCGTAGAGAGATTAATGCGCTTTTACGGCTTTC ATATGAATATCCTCCTTAG | pKD3 |
| *liv(KMGHF)* deletion | F:5’-CACATAAAAACAAAGCAACA CAACATCACGAATGGGGATT TTTGACTATGTGTAGGCTGG AGCTGCTTCG  R: 5’-CTTTTGACTCCCTATCAATC AACGTGT TATTACCCGCCTA AATACGCACTCATATGA ATA TCCTCCTTAG | pKD3 |
| *metN* deletion | F:5’-GCCCGTTTCAGGCATTCGAG ATGCCACGACTAACTTAATG ACGATAATAAATAATCAATG TGTAGGCTGGAGCTGCTTCG  R:5’-CGCCACGAACCAGCAGCCAC ATCATCGGCTCAGACATAAC CCAGTACCTCCATATGAATA TC CTCCTTAG | pKD3 |
| *yhaO-lacZ* transcriptional fusion | F: 5’-AAGTAACTGCAGCAGGGTTAACTGAAC  R: 5’-AATCAAGGTACCCATAATTTCTCGCTC | pSJ130  DH5a pir^+^/pSJ501 |
